# Alkaloids with Nitric Oxide Inhibitory Activities from the Roots of *Isatis tinctoria*

**DOI:** 10.3390/molecules24224033

**Published:** 2019-11-07

**Authors:** Dongdong Zhang, Yanhong Shi, Jingyi Li, Deqing Ruan, Qi Jia, Weiliang Zhu, Kaixian Chen, Yiming Li, Rui Wang

**Affiliations:** 1 School of Pharmacy, Shanghai University of Traditional Chinese Medicine, Shanghai 201203, China; zhangnatprod@163.com (D.Z.); jobrishua@163.com (J.L.); ruandeqing123@126.com (D.R.); q_jia@126.com (Q.J.); kxchen@mail.shcnc.ac.cn (K.C.); 2 Institute of TCM International Standardization of Shanghai University of Traditional Chinese Medicine, Shanghai 201203, China; shiyhtcm@shutcm.edu.cn; 3 Shanghai Institute of Materia Medica, Chinese Academy of Science, Shanghai 201203, China; wlzhu@simm.ac.cn

**Keywords:** *Isatis tinctoria* roots, alkaloids, structure identification, anti-inflammatory activity

## Abstract

As our ongoing research project on Ban Lan Gen (*Isatis tinctoria* roots), a total of 23 alkaloids were obtained. Compounds **1** and **2** contain an unusual C–C bond between the 2(1*H*)-quinolinone moiety and the phenol moiety and between the 2(1*H*)-quinolinone moiety and the 1*H*-indole moiety, respectively. Compound **3** possesses an unusual carbon skeleton and its putative biosynthetic pathway was discussed, and Compound **23** was deduced as a new indole alkaloid glycoside. Compounds **4–7** were identified as four new natural products by extensive spectroscopic experiments. Additionally, the anti-inflammatory activity was assessed based on nitric oxide (NO) production using Lipopolysaccharide-stimulated RAW264.7 macrophages. Compounds **9**, **15,** and **17** showed inhibitory effects with IC_50_ values of 1.2, 5.0, and 74.4 μM.

## 1. Introduction

**Figure 1:**
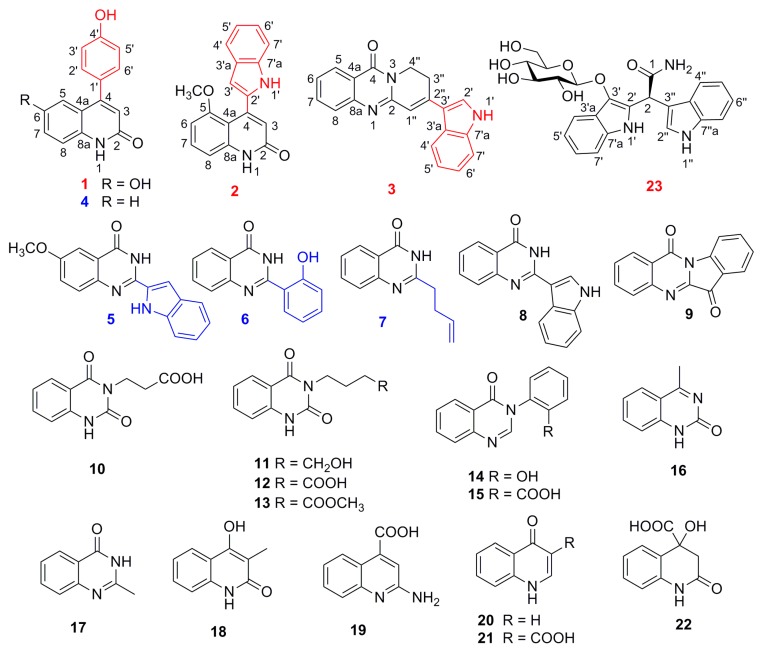
Structures of Compounds **1**–**23**.

*Isatis tinctoria* L. (synonym, *Isatis indigotica* Fort.), named Ban Lan Gen in the Chinese Pharmacopoeia, belongs to the gene *Isatis* (Brassicaceae family), which is widely distributed and cultivated in the North of the Yangtze River, China [1,2,3,4]. Alkaloids were considered as one of the characteristic constituents of this plant, which possess diverse bioactivities such as anti-inflammatory, antiviral, antibacterial, antitumor, and antioxidant activities [5,6,7]. Up to now, more than 100 alkaloids have been isolated from *I. tinctoria*, such as indole alkaloids, quinazolone alkaloids, quinoline alkaloids, and so on [1,2,3,4,5]. As our ongoing phytochemical and pharmacological research project on this plant [8,9,10,11,12], four new alkaloids and four new natural products, along with 15 known analogues, were obtained, and their structures and absolute configurations were determined by extensive spectroscopic data analysis, including one-dimensional and two-dimensional-NMR, HRESIMS, and IR, specific rotation data, and electronic circular dichroism (ECD) experiments. The known compounds (**4**–**22**, Figure 1) were identified by comparison of their spectroscopic and optical rotation data with those in the reported literature as 4-*p*-hydroxyphenyl-2(1*H*)-quinolinone (**4**) [13], 2-(1*H*-indol-2-yl)-6-methoxy-4(3*H*)-quinazolinone (**5**) [14], 2-(2-hydroxyphenyl)-4(3*H*)-quinazolinone (**6**) [15], 2-(but-3-en-1-yl)-4(3*H*)-quinazolinone (**7**) [16], 2-(1*H*-indol-2-yl)-4(3*H*)-quinolinone (**8**) [17], tryptanthrin (**9**) [18], 3-(2,4-dioxo-1,2- dihydroquinazolin-3(4*H*)-yl)propanoic acid (**10**) [19], indiforine C (**11**) [3], 4-(2,4-dioxo-1,2- dihydroquinazolin-3(4*H*)-yl)butanoic acid (**12**) [20], methyl 4-(2,4-dioxo-1,2-dihydroquinazolin- 3(4*H*)-yl)butanoate (**13**) [21], 3-(2-hydroxyphenyl)-4(3*H*)-quinazolinone (**14**) [22], 3-(2-carboxyphenyl)-4(3*H*)-quinazolinone (**15**) [23], 4-methyl-1,2-dihydro-2-oxoquinazoline (**16**) [24], 2-methyl-4(3*H*)-quinazolinone (**17**) [25], 4-hydroxy-3-methyl-2(1*H*)-quinolinone (**18**) [26], 2-amino-4-quinolinecarboxylic acid (**19**) [27], 4(1*H*)-quinolinone (**20**) [28], 4(1*H*)-quinolone-3- carboxylic acid (**21**) [29], and 1,2,3,4-tetrahydro-4-hydroxy-quinolinecarboxylic acid (**22**) [30]. The NO inhibitory activities of the isolates (**1**–**23**) were also evaluated against the LPS-stimulated RAW264.7 macrophages. In the present paper, we report the isolation and structure determination, putative biosynthetic pathway, and the NO inhibitory activities of these alkaloids.

## 2. Results and Discussion

**Figure 2:**
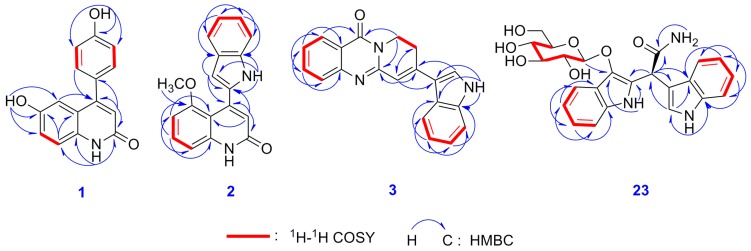
Key ^1^H-^1^H COSY and HMBC correlations of Compounds **1**–**3** and **23**.

Isatisindigoticanine E (**1**) was obtained as a yellow amorphous powder. The molecular formula was assigned as C_15_H_11_NO_3_ on the basis of the negative ion HRESIMS peak at *m/z* 252.0666 [M − H]^−^ (calculated 252.0666 [M − H]^−^), together with its one-dimensional-NMR data (Table 1). The ^1^H-NMR spectrum displayed signals of a 1,2,4-trisubstituted benzene ring [31] at [*δ*_H_ 7.20 (1H, d, *J* = 2.2 Hz, H-5), 6.63 (1H, dd, *J* = 8.3, 2.2 Hz, H-7), and 6.66 (1H, d, *J* = 8.3 Hz, H-8)], a 1,4 disubstituted benzene ring at [*δ*_H_ 7.57 (2H, d, *J* = 8.5 Hz, H-2’,6’) and 6.90 (2H, d, *J* = 8.5 Hz, H-3’, 5’) and also showed a trisubstituted double bond [9] at *δ*_H_ 7.48 (1H, s, H-3) and three exchangeable protons at *δ*_H_ 10.19 (1H, brs, NH-1), 10.12 (1H, brs, OH-6), and 8.96 (1H, brs, OH-4’). The ^13^C-NMR spectrum showed 15 carbon signals, among which 7 × C carbons at *δ*_C_ (169.5, 159.6, 152.2, 135.4, 126.1, 125.4, 122.5) and 8 × CH carbons at *δ*_C_ (136.7, 132.1, 132.1, 116.5, 116.1, 116.1, 110.7, 110.1) were found based on the DEPT 135 experiment. The two-dimensional-NMR spectroscopic features confirmed the inference above. The proton and protonated carbon resonances in the NMR spectra of **1** were unambiguously assigned by the HSQC experiments [32,33]. The ^1^H-^1^H COSY correlations (Figure 2) of H-2’,6’/H-3’,5’, along with HMBC correlations (Figure 2) of H-2’/C-4’ and H-3’/C-1’, indicated a phenol moiety in **1** [31]; ^1^H-^1^H COSY correlations of H-7/H-8, along with the HMBCs of NH-1/C-2, C-8, and C-8a, H-3/C-2, and C-4, and H-5/C-4 and C-7, indicated a 6-hydroxy-2(1*H*)-quinolinone moiety in **1** [34]; HMBCs of H-3/C-1’ and H-2’,6’/C-4 confirmed the 6-hydroxy-2(1*H*)-quinolinone moiety connected with the phenol moiety via a C-4-C-1’ bond. The structure of **1** was then determined, as depicted in Figure 1.

Isatisindigoticanine F (**2**) was obtained as a yellow amorphous powder. The molecular formula was assigned as C_1__8_H_1__4_N_2_O_2_ by the one-dimensional-NMR data and the HRESIMS positive ion peak at *m/z* 291.1125 [M + H]^+^ (calculated 291.1128 [M + H]^+^). The ^1^H-NMR spectrum (Table 1) of **2** showed signals of a 1,2,3-trisubstituted benzene ring at [*δ*_H_ 6.67 (1H, d, *J* = 7.5 Hz, H-6), 7.15 (1H, overlap, H-7) and 6.85 (1H, d, *J* = 7.3 Hz, H-8)], an *ortho*-disubstituted benzene ring at [*δ*_H_ 7.50 (1H, d, *J* = 7.5 Hz, H-4’), 7.15 (1H, overlap, H-5’), 7.00 (1H, dd, *J* = 7.5, 7.4 Hz, H-6’) and 7.14 (1H,overlap, H-7’)] [35]; two trisubstituted double bonds at *δ*_H_ 8.63 (1H, s, H-3) and 9.54 (1H, s, H-3’), as well as two exchangeable protons at *δ*_H_ 12.06 (1H, brs, NH-1) and 10.51 (1H, brs, NH-1’) and a methoxy group at *δ*_H_ 4.04 (3H, s, 5-OMe). After analysis of the ^13^C-NMR, DEPT 135 and HSQC data (Table 1), a 1*H*-indol-2-yl moiety (112.5, C; 133.5, CH; 126.3, C; 117.9, CH; 121.1, CH; 127.0, CH; 109.5, CH; 139.4, C) [8,10] and a 5-methoxy-2(1*H*)-quinolinone moiety (168.3, C; 130.6, CH; 118.8, C; 116.8, C; 155.0, C; 102.6, CH; 123.9, CH; 106.2, CH; 138.0, C; 55.9, CH_3_) were observed [34]. HMBCs of H-3/C-2’ and H-3’/C-4 indicated the 1*H*-indol-2-yl moiety connected with the 5-methoxy-2(1*H*)-quinolinone moiety via a C-4-C-2’ bond. These inferences were confirmed by detailed analysis of the two-dimensional-NMR data including HSQC, HMBC (Figure 2), and ^1^H–^1^H COSY (Figure 2) experiments. The structure of **2** was thus deduced, as depicted in Figure 1. 

Isatisindigoticanine G (**3**), a yellow amorphous powder, possessed the molecular formula of C_20_H_1__5_N_3_O based on the positive HRESIMS ion at *m/z* 314.1297 [M + H]^+^ (calculated 314.1288 [M + H]^+^) and one-dimensional-NMR data. The ^1^H-NMR spectrum (Table 1) of **3** showed signals of two *ortho*-disubstituted benzene rings at [*δ*_H_ 8.13 (1H, d, *J* = 8.0 Hz, H-5), 7.47 (1H, dd, *J* = 8.0, 7.2 Hz, H-6), 7.82 (1H, dd, *J* = 8.1, 7.2 Hz, H-7) and 7.72 (1H, d, *J* = 8.1 Hz, H-8)] and [*δ*_H_ 7.87 (1H, d, *J* = 7.5 Hz, H-4’),7.22 (1H, dd, *J* = 8.1, 7.5 Hz, H-5’), 7.25 (1H, dd, *J* = 8.1, 7.5 Hz, H-6’) and 7.50 (1H, d, *J* = 7.5 Hz, H-7’)], two trisubstituted double bonds at *δ*_H_ 7.84 (1H, d, *J* = 2.2 Hz, H-2’) and 8.13 (1H, s, H-1’’), as well as an exchangeable proton at *δ*_H_ 11.98 (1H, brs, NH-1’) [35]. The ^13^C-NMR and the DEPT 135 spectra (Table 1) displayed 8 × C carbons at *δ*_C_ (160.5, 156.8, 148.8, 136.4, 127.4, 125.6, 120.3, 112.5), 10 × CH carbons at *δ*_C_ (134.9, 128.3, 126.3, 126.2, 126.0, 123.0, 122.6, 120.9, 118.5, 112.6), and 2 × CH_2_ carbons at *δ*_C_ (44.7, 26.2). The two-dimensional-NMR spectra (Figure 2) of **3** showed the ^1^H-^1^H COSY correlations of H-5/H-6/H-7/H-8, H-3’’/H-4’’ and HMBCs from H-5/C-4 from H-1’’/C-2 and C-3’’ and from H-4’’/C-2 and C-4, which indicated a 8*H*-pyrido[2,1-b]-11(9*H*)-quinazolinone moiety in **3** [36]; ^1^H-^1^H COSY correlations of H-4’/H-5’/H-6’/H-7’ and the HMBCs from NH-1’/C-2’, C-3’, C-3’a, and C-7’a indicated a 1*H*-indol-3-yl moiety in **3** [10]. HMBCs from NH-1’/C-9 and C-6, and from H-2’/C-2’’ and H-1’’/C-3’ determined the 8*H*-pyrido[2,1-b]-11(9*H*)-quinazolinone moiety connected with the 1*H*-indol-3-yl moiety via a C-2’’-C-3’ bond. The structure of **3** was thus determined, as depicted in Figure 1.

**Figure 3:**
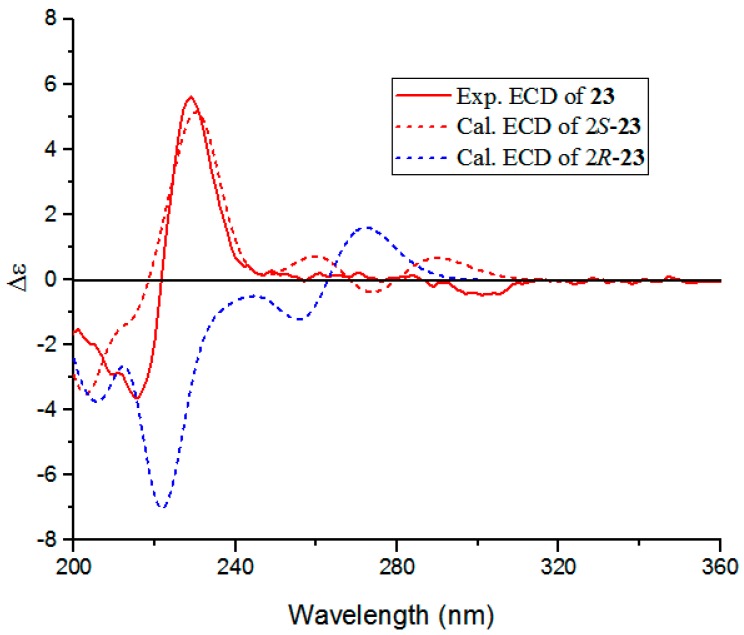
Experimental and calculated ECD spectra of **23**.

Isatindigoside D (**23**) was isolated as a red amorphous powder with [α]${}_{D}^{20}$ + 12.1° (*c* 0.19, MeOH). Its molecular formula of C_23_H_22_N_2_O_7_ (14 IHD) was deduced from the NMR data and the HRESIMS positive ion peak at *m*/*z* 490.1592 [M + Na]^+^, (calculated 490.1585 [M + Na]^+^). When comparing the one-dimensional (Table 2) and two-dimensional-NMR data (Figure 2) with the reported bisindoloside of isatindigobisindoloside C [35], they showed almost identical NMR spectroscopic features except for the differences around C-2 (downfield of C-2’, C-3’, and C-3’’, upfield of C-2’’). These differences, along with the optical rotation data ([α]${}_{D}^{20}$ + 12.1, *c* 0.19 in MeOH) supported Compound **23,** would be the C2-epimer of isatindigobisindoloside C ([α]${}_{D}^{20}$ − 33.9, *c* 0.11 in MeOH) [35]. The experimental and calculated ECD curves of (2*S*)-**23** matched well (Figure 3), which confirmed the *S* absolute configuration of **23** [35,37]**,** and the calculation details are listed in the Supporting Information (Figures S33 and S34). Acid hydrolysis of **23** resulted in the product of d-glucose, which was confirmed by GC analysis of the acetylation derivative of the hydrolysate of **23** and the authentic sugars (*t*_R_ d-glucose 45.23 min, *t*_R_ l-glucose 45.38 min) [8,9]. The large coupling constant of Glc-H1 (*J* = 7.8 Hz) revealed the β-glucopyranosyl linkage in **23** [38,39]. Accordingly, the structure of isatindigoside D (**23**) was elucidated as depicted (Figure 1).

NO is a messenger molecule that is widespread in cells and can affect a variety of physiological and pathological processes. The production of NO causes tissue damage and can trigger a variety of inflammatory diseases. LPS induces the release of NO from RAW264.7 cells by detecting the release of NO widely used to investigate the anti-inflammatory effects of the compounds [2,10,40]. As our ongoing phytochemical and pharmacological research project on *I. tinctoria* [8,9,10,11,12], Compounds **1**–**23** were obtained and were evaluated for their anti-inflammatory activity based on NO inhibitory effects in the LPS-activated RAW 264.7 cells [40]. The cytotoxicity of Compounds **1**–**23** were tested at three different concentrations (25, 50, and 100 μM), and the results showed that only Compound **9** showed cytotoxicity above 25 μM, while the other compounds were above 100 μM. The results of NO production showed that Compounds **9**, **15**, and **17** exhibited inhibitory activities with IC_50_ values of 1.2, 5.0, and 74.4 μM (Table 3).

**Figure 4:**
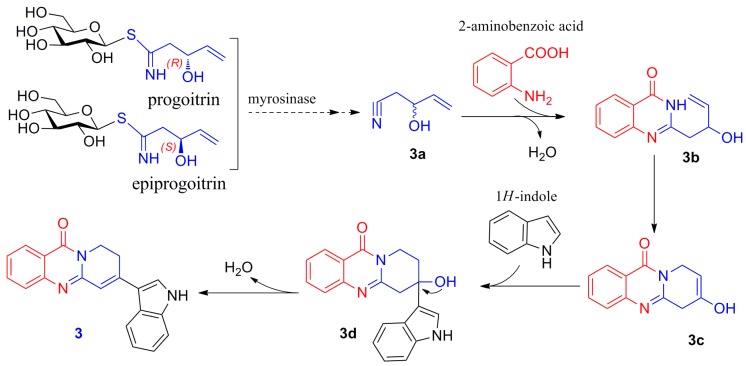
Putative biosynthetic pathway of **3**.

Isatisindigoticanine G (**3**) is the first example of a 8*H*-pyrido[2,1-b]-11(9*H*)-quinazolinone moiety connected with a 1*H*-indol-3-yl moiety via a C–C bond of C-2’’–C-3’. For its unusual structural features, a plausible biosynthetic pathway is discussed in Figure 4. First, myrosinase catalyzed hydrolysis of progoitrin and epiprogoitrin to give **3a** [1]. **3a** was connected with 2-aminobenzoic acid moiety by steps of dehydration to give **3b** [10], and then **3c** was obtained via a cyclization reaction of **3b** [2,11]. **3c** was connected with 1*H*-indole moiety by enzyme-catalyzed reaction to give **3d** [5] and was then changed via a dehydration reaction to give **3** [9,10,11].

## 3. Experimental Section

The General Experimental Procedures, Extraction and Isolation, Plant Materials, Inhibitory Assay of NO Production and ECD Calculation sections are listed in the Supporting Information.

### 3.1. Physical and Spectroscopic Data of Isatisindigoticanines E–G and Isatindigoside D

Isatisindigoticanine E (**1**), a yellow amorphous powder; IR (KBr) *ν*_max_: 3406, 2923, 1647, 1609, 1556, 1517, 1466, 1383, 1273, 1093, 745 cm^−1^; *m/z* 356.1398 [M + H]^+^ (calculated 356.1394 [M + H]^+^); ^1^H-NMR (DMSO-*d*_6_, 600 MHz) and ^13^C-NMR (DMSO-*d*_6_, 150 MHz); see Table 1.

Isatisindigoticanine F (**2**), a yellow amorphous powder; IR (KBr) *ν*_max_: 3456, 1679, 1621, 1516, 1461, 1319, 1206, 1135, 1021, 952, 749 cm^−1^; *m/z* 291.1125 [M − H]^−^ (calculated 291.1128 [M + H]^+^); ^1^H-NMR (DMSO-*d*_6_, 600 MHz) and ^13^C-NMR (DMSO-*d*_6_, 150 MHz); see Table 1.

Isatisindigoticanine G (**3**), a yellow amorphous powder; IR (KBr) *ν*_max_: 3404, 2919, 1708, 1601, 1468, 1400, 1384, 1092, 745 cm^−1^; *m/z* 314.1297 [M + H]^+^ (calculated 314.1288 [M + H]^+^); ^1^H-NMR (DMSO-*d*_6_, 600 MHz) and ^13^C-NMR (DMSO-*d*_6_, 150 MHz); see Table 1.

Isatindigoside D (**23**), a red amorphous powder; [α]${}_{D}^{20}$ + 12.1 (*c* 0.19, MeOH); IR (KBr) *ν*_max_: 3420, 2939, 1722, 1598, 1514, 1461, 1261, 1069, 1025, 859, 813 cm^−1^; HRESIMS: *m*/*z* 490.1592 [M + Na]^+^, (calculated 490.1585 [M + Na]^+^); ^1^H and ^13^C-NMR (600 and 150 MHz in DMSO-*d*_6_); see Table 2.

### 3.2. Absolute Configuration Determination of Sugar

Compound **23** (2 mg) was hydrolyzed in 2 M hydrochloric acid (4 mL) at 80 °C for 2 h. After cooling, the solution was concentrated under vacuum, dissolved with water, and extracted twice with dichloromethane (CH_2_Cl_2_). The residue was dissolved in distilled water and reduced with NaBH_4_ for 3 h at room temperature. After neutralization with AcOH and evaporation to dryness, the residue was acetylated with Ac_2_O for 1 h at 100 °C. The resulting alditol acetate was subjected to GC analysis under the following conditions: capillary column, HP-5ms (60 m × 0.25 mm × 0.25 μm); detector, FID; detector temperature, 280 °C; injection temperature, 280 °C; initial temperature 140 °C, subsequently increased to 240 °C at a rate of 5 °C/min, and then 1 min to increase to 260 °C, finally, subsequent increase to 280 °C at a rate of 2 °C/min; carrier, N_2_ gas [8,9]. The D glucose moiety in **23** was confirmed by the comparison of their retention times (*t*_R_) with those of authentic sugars (*t*_R_ d-glucose 45.23 min, *t*_R_ l-glucose 45.38 min).

## 4. Conclusions

In this paper, a total of 23 alkaloids were reported, including four new ones: isatisindigoticanines E–G (**1**–**3**) and isatindigoside D (**23**). Four new natural products and 15 known analogues were isolated from Ban Lan Gen. Isatisindigoticanine G possesses an unusual carbon skeleton of an 8*H*-pyrido[2,1-b]-11(9*H*)-quinazolinone moiety connected with a 1*H*-indole moiety via a C–C bond of C-2’’–C-3’. Compounds **9**, **15**, and **17** showed NO inhibitory effects with IC_50_ values of 1.2, 5.0, and 74.4 μM in the LPS-stimulated RAW264.7 macrophages. This study is important as it explains the chemical and biological diversity of Ban Lan Gen. Furthermore, the new structures need more biocativity experiments to discover their more meaningful uses, which may stimulate us to better develop and utilize these compounds.

## Figures and Tables

**Table 1:** ^1^H-NMR (600 MHz in DMSO-*d*_6_) and ^13^C-NMR data (150 MHz in DMSO-*d*_6_) of **1**–**3**.

No.	1		2		3	
	*δ*H	*δ*C	*δ*H	*δ*C	*δ*H	*δ*C
1	10.19, s		12.06, brs			
2		169.5		168.3		156.8
3	7.48, s	136.7	8.63, s	130.6		
4		126.1		118.8		160.5
4a		122.5		116.8		120.3
5	7.20, d (2.2)	110.1		155.0	8.13, d (8.0)	126.3
6		152.2	6.67, d (7.5)	102.6	7.47, dd (8.0, 7.2)	126.0
7	6.63, dd (8.3, 2.2)	110.7	7.15, overlap	123.9	7.82, dd (8.1, 7.2)	134.9
8	6.66, d (8.3)	116.5	6.85, d (7.3)	106.2	7.72, d (8.1)	126.2
8a		135.4		138.0		148.8
1’		125.4	10.51, s		11.98, brs	
2’	7.57, d (8.5)	132.1		112.5	7.84, d (2.2)	128.3
3’	6.90, d (8.5)	116.1	9.45, s	133.5		112.5
3’a				126.3		127.4
4’		159.6	7.50, d (7.5)	117.9	7.87, d (7.5)	118.5
5’	6.90, d (8.5)	116.1	7.15, overlap	121.1	7.22, dd (8.1, 7.5)	120.9
6’	7.57, d (8.5)	132.1	7.00, dd (7.5, 7.4)	127.0	7.25, dd (8.1, 7.5)	123.0
7’			7.14, overlap	109.5	7.50, d (7.5)	112.6
7’a				139.4		136.4
1’’					8.13, s	122.6
2’’						125.6
3’’					3.17, 2H, m	26.2
4’’					4.25, 2H, t (7.0)	44.7
OMe			4.04, s	55.9		
6-OH	10.12, s					
4’-OH	8.96, s					

**Table 2:** ^1^H-NMR (600 MHz in DMSO-*d*_6_) and ^13^C-NMR data (150 MHz in DMSO-*d*_6_) of **23.**

No.	23		No.	23	
	*δ*_H_ (*J* in Hz)	*δ*_C_		*δ*_H_ (*J* in Hz)	*δ*_C_
1a	7.45, brs	172.9	3’’		112.1
1b	7.14, brs		3’’a		126.7
2	5.68, s	38.7	4’’	7.56, d (8.0)	118.4
1’	10.35, brs		5’’	6.93, dd (8.0, 7.1)	118.9
2’		126.8	6’’	7.04, dd (8.1, 7.1)	120.5
3’		133.0	7’’	7.32, d (8.1)	111.4
3’a		121.2	7’’a		136.0
4’	7.77, d (8.0)	118.1	Glc-1	4.63, d (7.8)	106.6
5’	6.92, dd (8.0, 7.2)	118.4	2	3.38, overlap	74.1
6’	6.97, dd (8.1, 7.2)	120.8	3	3.26, m	76.8
7’	7.26, d (8.1)	111.6	4	3.28, m	69.8
7’a		133.3	5	3.14, m	77.2
1’’	10.94, brs		6a	3.56, dd (10.8, 5.6)	61.0
2’’	7.38, s	123.9	6b	3.67, dd (10.8, 1.8)	

**Table 3:** NO inhibitory activities of Compounds **1**–**23** in RAW 264.7 cell line.

Compounds	IC_50_^a^	Cytotoxicity	Compounds	IC_50_^a^	Cytotoxicity
**1**	>100	>100	**1** **3**	>100	>100
**2**	>100	>100	**1** **4**	>100	>100
**3**	>100	>100	**1** **5**	5.0 ± 1.3	>100
**4**	>100	>100	**16**	>100	>100
**5**	>100	>100	**17**	74.4 ± 3.8	>100
**6**	>100	>100	**18**	>100	>100
**7**	>100	>100	**19**	>100	>100
**8**	>100	>100	**2** **0**	>100	>100
**9**	1.2 ± 0.9	>25	**2** **1**	>100	>100
**10**	>100	>100	**2** **2**	>100	>100
**11**	>100	>100	**23**	>100	>100
**12**	>100	>100	AG ^b^	22.7 ± 0.4	>100

^a^ IC_50_ values were expressed as mean ± SD (*n* = 3). ^b^ AG = aminoguanidine hydrochloride was used as the positive control.

## Supplementary Materials

The following are available online https://www.mdpi.com/1420-3049/24/22/4033/s1, Copies of IR, HREIMS, ^1^H-NMR, ^13^C-NMR, DEPT 135, HSQC, HMBC, and ^1^H-^1^H COSY of **1**–**3** and **23**. Experimental and calculated ECD spectra of **23**.
