# Supplementary material for: Alkaloids with Nitric Oxide Inhibitory Activities from the Roots of Isatis tinctoria
Source: Molecules. 2019 Nov 7;24(22):4033. doi: 10.3390/molecules24224033 (PMC6891263; doi:10.3390/molecules24224033)
Supplement: Supplementary file 1 [file molecules-24-04033-s001.pdf]

Supplementary Materials

Alkaloids with Nitric Oxide Inhibitory Activities from the Roots *Isatis tinctoria*

Dongdong Zhang [1,#], Yanhong Shi [2,#], Kang Du [1], Deqing Ruan [1], Qi Jia [1], Kaixian Chen [1,3],

Yiming Li [1,*] and Rui Wang [1,*]

Affiliation

[1] School of Pharmacy, Shanghai University of Traditional Chinese Medicine, Shanghai, 201203, China

[2] Institute of TCM International Standardization of Shanghai University of Traditional Chinese Medicine, Shanghai, 201203, China

[3] Shanghai Institute of Materia Medica, Chinese Academy of Science, Shanghai, 201203, China

[#] These authors contribute equally to this work.

Corresponding Author

[*] E-mail: wr@shutcm.edu.cn (Rui Wang), Tel: 86 21 51322181, Fax: 86 21 51322193.

E-mail: ymlius@163.com (Yiming Li), Tel: 86 21 51322191, Fax: 86 21 51322193.

General experimental procedures

The HR-ESI-MS was performed using a Q-TOF-Ultima mass spectrometer (Milford, MA, USA); ECD spectra were obtained on a Applied photophysics brighttime chirascan (AppliedPhotophysics, UK); IR spectra were recorded on a Nicolet iS10 instrument (Thermo Fisher Scientific, USA); 1D and 2D NMR spectra were recorded on a Bruker-Avance 600 or Bruker-Avance 400 instrument (Bruker, Germany); Optical rotation was measured using a Rudolph Autopol VI polarimeter (Rudolph, USA); Semipreparative HPLC was performed on an Agilent infinity II system equipped with a DAD detector (Agilent, USA) and a Capcell Pak C_{18} column (10 mm × 250 mm, 5μm particles, Shiseido, Japan); Sephadex LH-20 (GE Healthcare Bio-Sciences AB); Reversed-phase C_{18} silica gel 5 μm, YMC Co., Ltd. Japan); MCI gel (CHP-20 P, Mitsubishi Chemical Industries Co., Ltd. Japan); Silica gel (100–200 mesh and 200–300 mesh; Qingdao Haiyang Chemical, China); All solvents used in CC were of analytical grade (Sinopharm Chemical Reagent Co., Ltd. China). Murine macrophage RAW 264.7 cells were purchased from the Cell Bank of Chinese Academy of Sciences (Shanghai, China); Multiskan go Microplate Reader (Thermo Fisher Scientific, USA); Thermo HERH Cell incubator (Thermo Fisher Scientific, USA); Heal Force bechtop (Thermo Fisher Scientific, USA); Pipettor (Eppendorf, German); *p*-aminobenzenesulfonamide (Sigma-Alorich: #SLBC0284V); N-1-Naphthylethylenediamine Dihydrochloride (Sigma-Alorich: #SLBC1304V); aminoguanidine hydrochloride (AH, Sigma-Alorich).

Plant Material

The *Isatis tinctoria* roots (Ban Lan Gen) was collected on July, 2017 from the Daqing city, HeiLongJiang Province of China, and it was authenticated by one of our Co-authors Rui Wang professor (School of Pharmacy, Shanghai University of Traditional Chinese Medicine). A voucher specimen (herbarium No. 20170716SL) has been deposited in the Medicinal Plants Herbarium (MPH), School of Pharmacy, Shanghai University of Traditional Chinese Medicine, Shanghai, China.

Extraction and isolation

The air-dried and pulverized root of *I. tinctoria* (synonym *Isatis indigotica* Fort., 45 kg) was extracted with 80% EtOH under reflux three

times. After removing the solvent under reduced pressure, the concentrated residue was successively partitioned with petroleum ether (PE), dichloromethane (CH_2Cl_2) and *n*-BuOH. The CH_2Cl_2 extract (170 g) was subjected to column chromatography (CC) on silica gel, eluting with a gradient solvent system (CH_2Cl_2-MeOH, 100:0 − 100:20) to give eleven fractions (F1 − F11); F3 (16 g) was subjected to CC on silica gel, eluting with (CH_2Cl_2-MeOH, 100:1 − 100:5) to give six subfractions (F3-1 − F3-6). F3-1 (1.9 g) was subjected to CC on Sephadex LH-20 gel, eluting with (CH_2Cl_2-MeOH, 1:1) and then purified by HPLC with MeCN-H_2O (42:58) to afford **6** (3.7 mg; t_R = 26.8 min) and **7** (5.2 mg; t_R = 28.3 min); F3-2 (1.7 g) was subjected to CC on Sephadex LH-20 gel, eluting with (CH_2Cl_2-MeOH, 1:1) and then purified by HPLC with MeCN-H_2O (38:62) to afford **4** (4.1 mg; t_R = 20.7 min); F3-3 (0.9 g) was subjected to CC on Sephadex LH-20 gel, eluting with (CH_2Cl_2-MeOH, 1:1) and then purified by HPLC with MeCN-H_2O (32:68) to afford **2** (5.0 mg; t_R = 21.3 min); F3-4 (2.6 g) was subjected to CC on Sephadex LH-20 gel, eluting with (CH_2Cl_2-MeOH, 1:1) and then purified by HPLC with MeCN-H_2O (30:70) to afford **3** (3.8 mg; t_R = 18.6 min), **5** (4.5 mg; t_R = 27.4 min) and **8** (3.9 mg; t_R = 20.9 min); F-4 (14 g) was subjected to CC on silica gel, eluting with (CH_2Cl_2-MeOH, 100:1 ~ 100:5) to give five subfractions (F4-1 − F4-5). F4-3 (1.9 g) was subjected to CC on Sephadex LH-20 gel, eluting with (CH_2Cl_2-MeOH, 1:1) and then purified by HPLC with MeCN-H_2O (25:75) to afford **16** (4.1 mg, t_R = 28.3 min) and **17** (6.2 mg, t_R = 21.1 min); F4-5 (2.3 g) was subjected to CC on Sephadex LH-20 gel, eluting with (CH_2Cl_2-MeOH, 1:1) and then purified by HPLC with MeCN-H_2O (20:80) to afford **14** (16.4 mg; t_R = 33.7 min); F5 (7g) was subjected to CC on silica gel, eluting with (CH_2Cl_2-MeOH, 100:2 ~ 100:10) to give six subfractions (F5-1 − F5-6). F5-5 (0.5 g) was purified by HPLC with MeCN-H_2O (26:74) to afford **1** (3.4 mg, t_R = 18.2 min) and **20** (8.4 mg, t_R = 21.0 min); F5-6 (0.7 g) was purified by HPLC with MeCN-H_2O (23:77) to afford **11** (3.0 mg, t_R = 16.8 min) and **13** (13.2 mg, t_R = 15.1 min). F6 (8 g) was subjected to CC on silica gel, eluting with (CH_2Cl_2-MeOH, 100:2 ~ 100:10) to give seven subfractions (F6-1 − F6-7). F6-6 (0.6 g) was purified by HPLC with MeCN-H_2O (18:82) to afford **10** (2.8 mg, t_R = 19.2 min), **12** (3.8 mg, t_R = 23.6 min) and **23** (5.2 mg, t_R = 20.5 min); F6-7 (0.6 g) was purified by HPLC with MeCN-H_2O (20:80) to afford **15** (3.3 mg, t_R = 14.2 min), **18** (3.5 mg, t_R = 16.0 min) and **21** (5.5 mg, t_R = 17.4 min). F7 (5g) was subjected to CC on silica gel, eluting with (CH_2Cl_2-MeOH, 100:5 ~ 100:10) to give four subfractions (F7-1 − F7-4). F7-1 (0.2g) was purified by HPLC with

MeCN-H_2O (19:81) to afford **9** (11.2 mg, t_R = 36.6 min). F8 (4g) was subjected to CC on RP-C_{18} eluting with MeCN-H_2O (10%, 30%, 60%) to give three subfractions (F8-1 − F8-3). F8-1 (0.4 g) was purified by HPLC with MeCN-H_2O (24:76) to afford **19** (3.4 mg, t_R = 8.3 min) and **22** (2.6 mg, t_R = 9.6 min).

Inhibitory Assay of NO Production

Cytotoxicity was examined using the Cell Counting Kit-8 (CCK-8). RAW264.7 cells were taken from logarithmic growth stage and the cell density reached above 80%. And then, the cells were inoculated on 96-well cell culture plates at 100 microns/well, with 3×10^4 cells per well, cultured overnight at 37 °C with 5% CO_2. After 24h, the culture medium in the 96-well plate was discarded, the solution was changed and different concentrations (25 μM, 50 μM and 100 μM) of drugs were added. The absorbance at 450 nm was measured using a microplate reader (Thermo Fisher Scientific). Viability was defined as the ratio (expressed as a percentage) of absorbance values of treated cells to untreated cells. The results were listed in Table 3.

Compounds **1**－**23** were dissolved in dimethyl sulfoxide (DMSO) and diluted with complete medium to 6 degrees of concentration (Compound **9**: 0.1, 0.5, 1, 5, 10 and 25 μM, while the others: 0.1, 1, 5, 25, 50 and 100 μM) for inhibition rate determination. RAW 264.7 cells were maintained in Dulbecco's modified Eagle's medium (high-glucose condition) supplemented with 10% fetal calf serum, 100 U/mL penicillin, and 100 μg/mL streptomycin at 37 °C in 5% CO_2. RAW 264.7 cells were pretreated with each tested compound for 30 min, and then stimulated with lipopolysaccharide (LPS) (100 ng/mL) for 24 h. Aminoguanidine hydrochloride (100 μM) was used as a positive control. The NO production was measured using the Griess reagent. Briefly, the cell culture supernatant was reacted with equal volumes of Griess reagent in a 96-well plate for 10 min, and then the absorbance at 540 nm was measured by a plate reader. All experiments were performed in triplicate. All tested compounds were prepared as stock solutions with a concentration of 10 mM in DMSO. The IC_{50} values of compounds **1**－**23** were calculated.

ECD calculation of compound 23

The conformers of compound **23** were obtained using the MM2 force field with ChemBio3D software. Gaussian 09 software was utilized for the semiempirical PM3 quantum mechanical geometry optimizations and the time-dependent density functional theory (TDDFT) ECD was calculated at the b3lyp/6-31g(d) level. The ECD spectra conformers of compound **23** were obtained using SpecDis 1.62 and compared with the experimental data.

List of Content

No.	Content	page
1	**Figure S1.** The IR spectrum of **1** (in KBr)	**S8**
2	**Figure S2.** The HR-ESI-MS spectrum of **1** (in MeOH)	**S9**
3	**Figure S3.** The ^{1}H NMR spectrum of **1** (in DMSO-d_6)	**S10**
4	**Figure S4.** The ^{13}C NMR spectrum of **1** (in DMSO-d_6)	**S11**
5	**Figure S5.** The DEPT 135 spectrum of **1** (in DMSO-d_6)	**S12**
6	**Figure S6.** The HSQC spectrum of **1** (in DMSO-d_6)	**S13**
7	**Figure S7.** The HMBC spectrum of **1**(in DMSO-d_6)	**S14**
8	**Figure S8.** The ^{1}H-^{1}H COSY spectrum of **1** (in DMSO-d_6)	**S15**
9	**Figure S9.** The IR spectrum of **2** (in KBr)	**S16**
10	**Figure S10.** The HR-ESI-MS spectrum of **2** (in MeOH)	**S17**
11	**Figure S11.** The ^{1}H NMR spectrum of **2** (in DMSO-d_6)	**S18**
12	**Figure S12.** The ^{13}C NMR spectrum of **2** (in DMSO-d_6)	**S19**
13	**Figure S13.** The DEPT 135 spectrum of **2** (in DMSO-d_6)	**S20**
14	**Figure S14.** The HSQC spectrum of **2** (in DMSO-d_6)	**S21**
15	**Figure S15.** The HMBC spectrum of **2** (in DMSO-d_6)	**S22**
16	**Figure S16.** The ^{1}H-^{1}H COSY spectrum of **2** (in DMSO-d_6)	**S23**
17	**Figure S17.** The IR spectrum of **3** (in KBr)	**S24**
18	**Figure S18.** The HR-ESI-MS spectrum of **3** (in MeOH)	**S25**
19	**Figure S19.** The ^{1}H NMR spectrum of **3** (in DMSO-d_6)	**S26**
20	**Figure S20.** The ^{13}C NMR spectrum of **3** (in DMSO-d_6)	**S27**
21	**Figure S21.** The DEPT 135 spectrum of **3** (in DMSO-d_6)	**S28**
22	**Figure S22.** The HSQC spectrum of **3** (in DMSO-d_6)	**S29**

23	**Figure S23.** The HMBC spectrum of **3** (in DMSO-d_6)	**S30**
24	**Figure S24.** The ^{1}H-^{1}H COSY spectrum of **3** (in DMSO-d_6)	**S31**
25	**Figure S25.** The ROESY spectrum of **3** (in DMSO-d_6)	**S32**
26	**Figure S26.** The IR spectrum of **23** (in KBr)	**S33**
27	**Figure S27.** The HR-ESI-MS spectrum of **23** (in MeOH)	**S34**
28	**Figure S28.** The ^{1}H NMR spectrum of **23** (in DMSO-d_6)	**S35**
29	**Figure S29.** The ^{13}C NMR spectrum of **23** (in DMSO-d_6)	**S36**
30	**Figure S30.** The DEPT 135 spectrum of **23** (in DMSO-d_6)	**S37**
31	**Figure S31.** The HSQC spectrum of **23** (in DMSO-d_6)	**S38**
32	**Figure S32.** The HMBC spectrum of **23** (in DMSO-d_6)	**S39**
33	**Figure S33.** b3lyp/6-31g(d) optimized lowest energy conformers for (2*S*)-**23** and their equilibrium populations	**S40**
34	**Figure S34.** b3lyp/6-31g(d) optimized lowest energy conformers for (2*R*)-**23** and their equilibrium populations	**S41**
35	**Figure S35**. Experimental and calculated ECD spectrum of **23**	**S42**

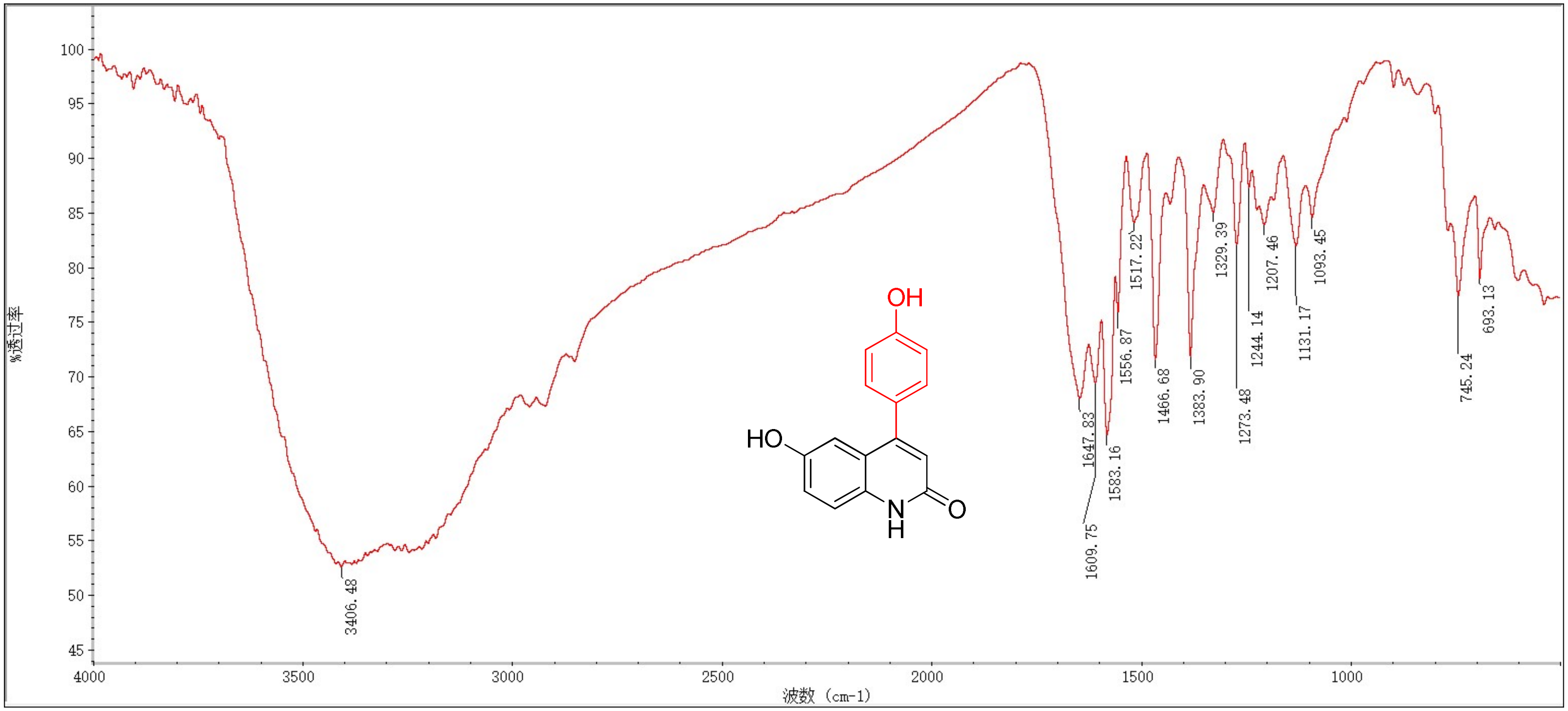

Figure S1. The IR spectrum of **1** (in KBr)

Qualitative Analysis Report

**Data Filename**	ESIH_20181116_ZWL_ZYT_N_15-1.d	**Sample Name**	ZYT-002-46
**Sample Type**	Sample	**Position**	P1-C7
**Instrument Name**	Agilent G6520 Q-TOF	**Acq Method**	20160324_MS_ESIH_NEG_1min.m
**Acquired Time**	11/16/2018 16:44:16	**IRM Calibration Status**	Success
**DA Method**	small molecular data analysis method.m	**Comment**	ESIH by ZZY

User Spectra

Fragmentor Voltage	Collision Energy	Ionization Mode
110	0	ESI

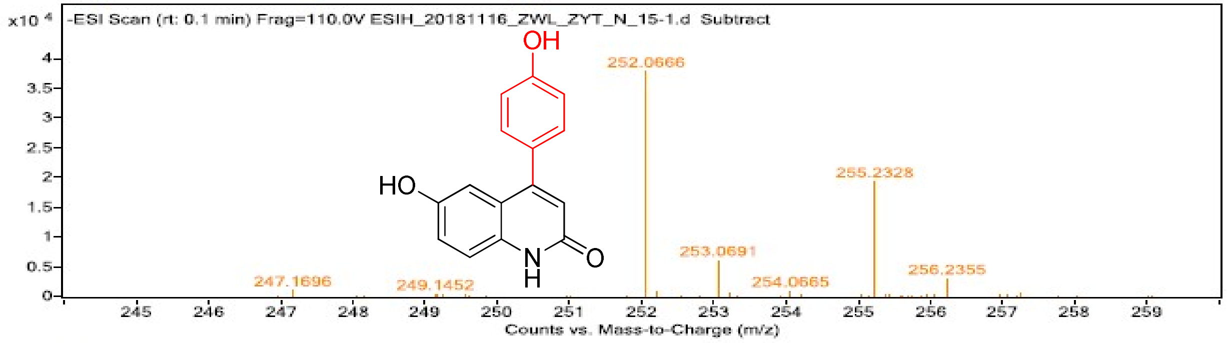

Formula Calculator Results

m/z	Calc m/z	Diff (mDa)	Diff (ppm)	Ion Formula	Ion
252.0666	252.0666	0.01	0.05	C15 H10 N O3	(M-H)-

--- End Of Report ---

Figure S2. The HR-ESI-MS spectrum of **1** (in MeOH)

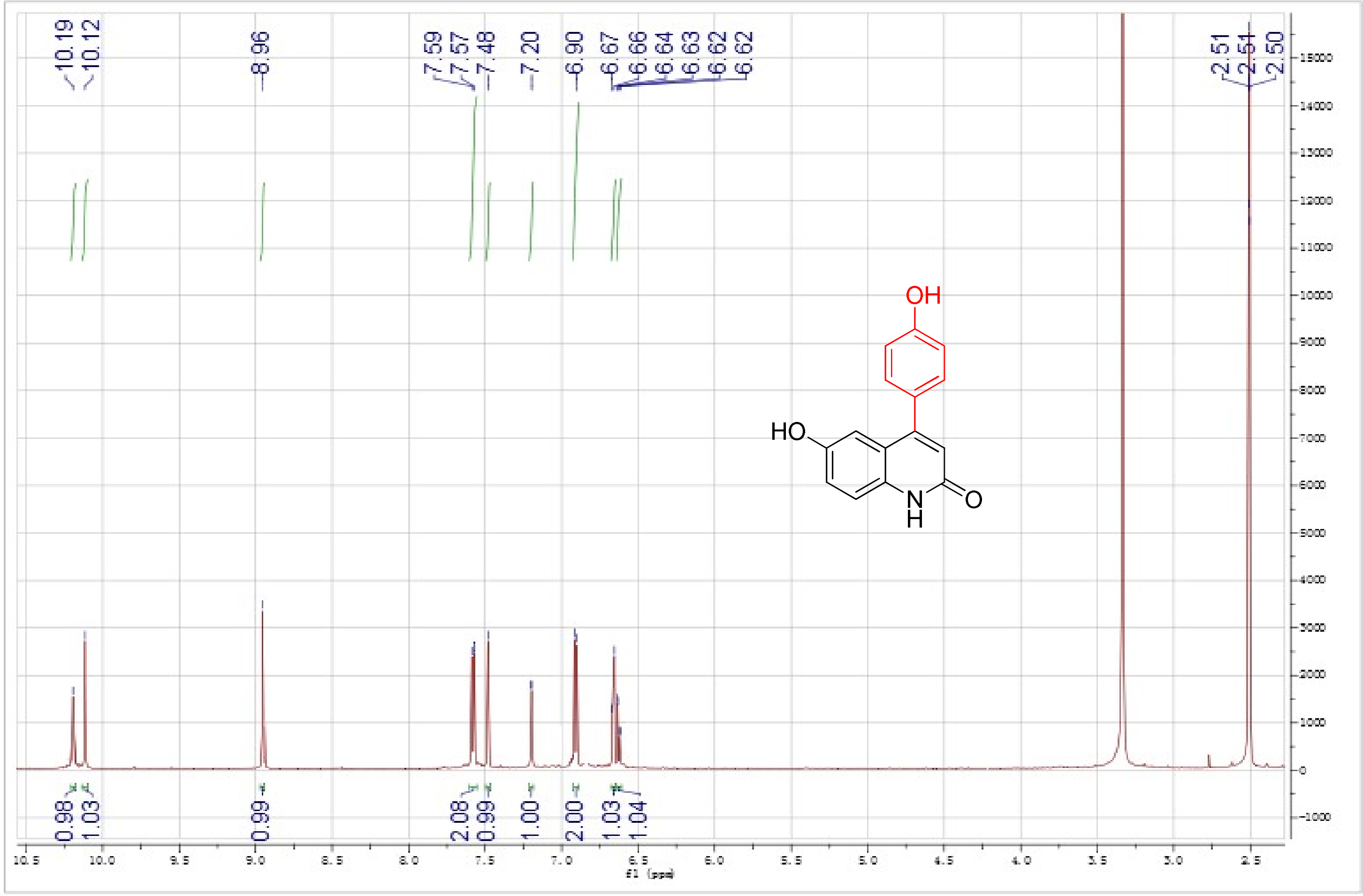

Figure S3. The ^{1}H NMR spectrum of **1** (in DMSO-d_6)

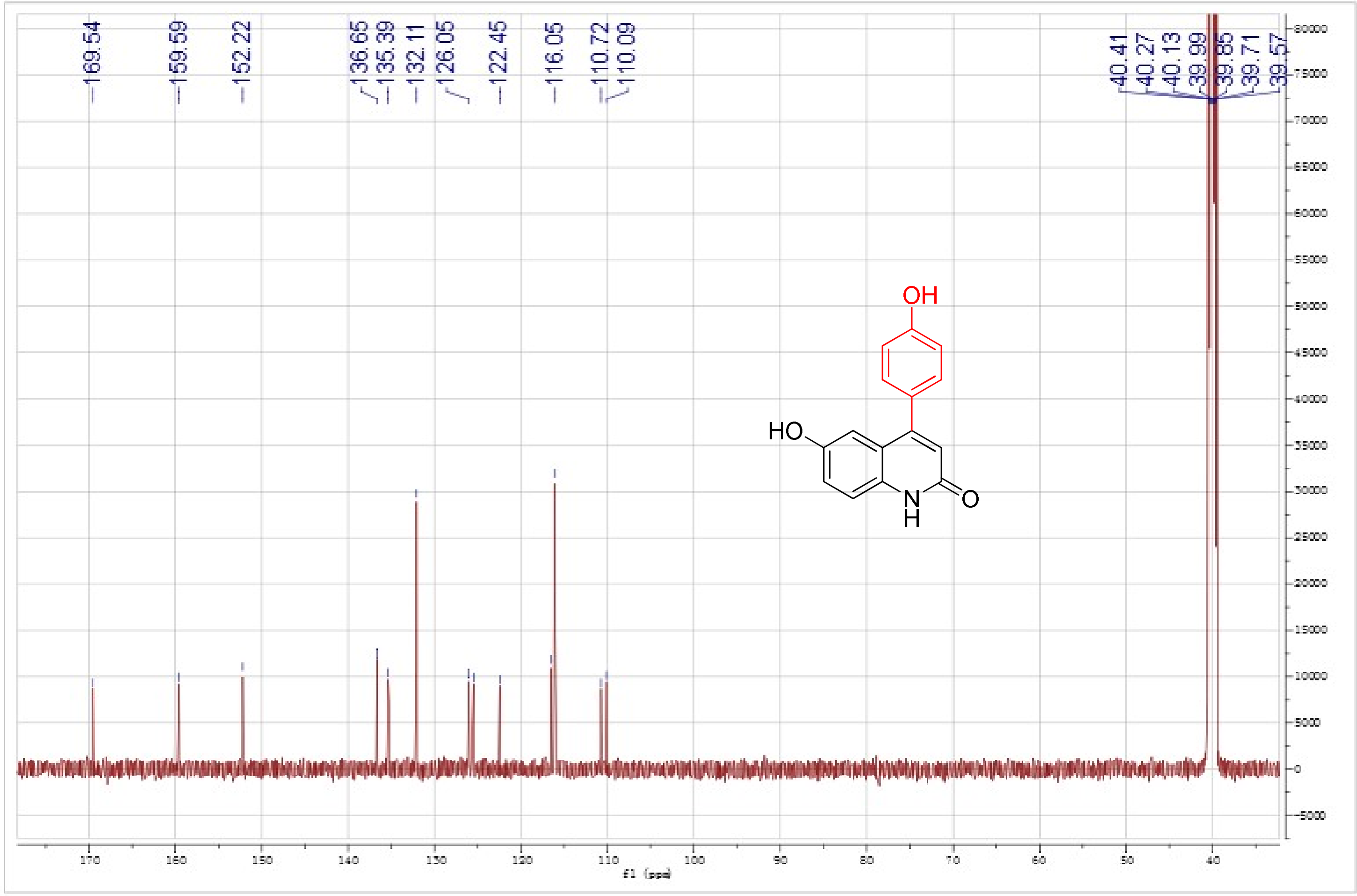

Figure S4. The ^{13}C NMR spectrum of **1** (in DMSO-d_6)

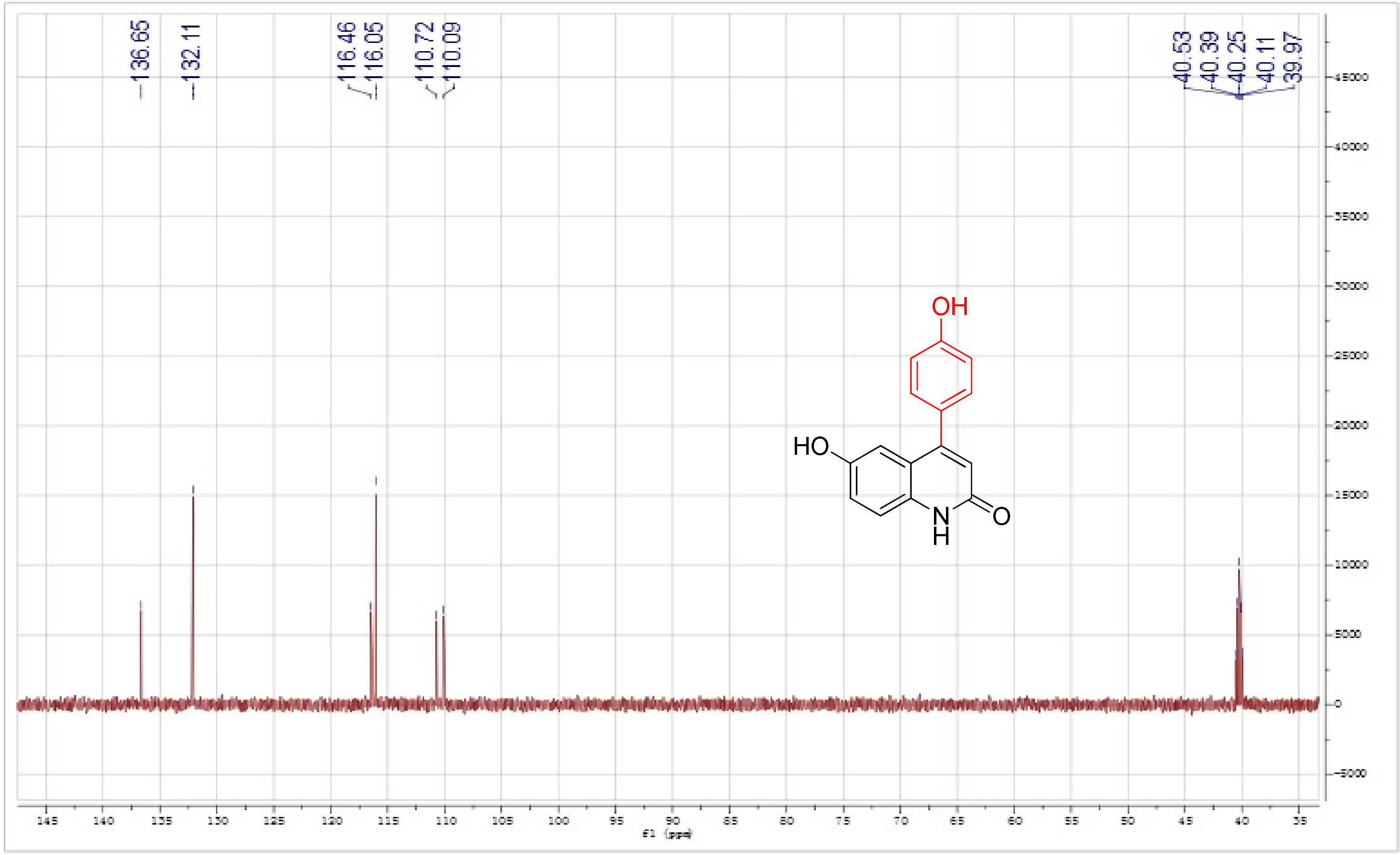

Figure S5. The DEPT 135 spectrum of **1** (in DMSO-d_6)

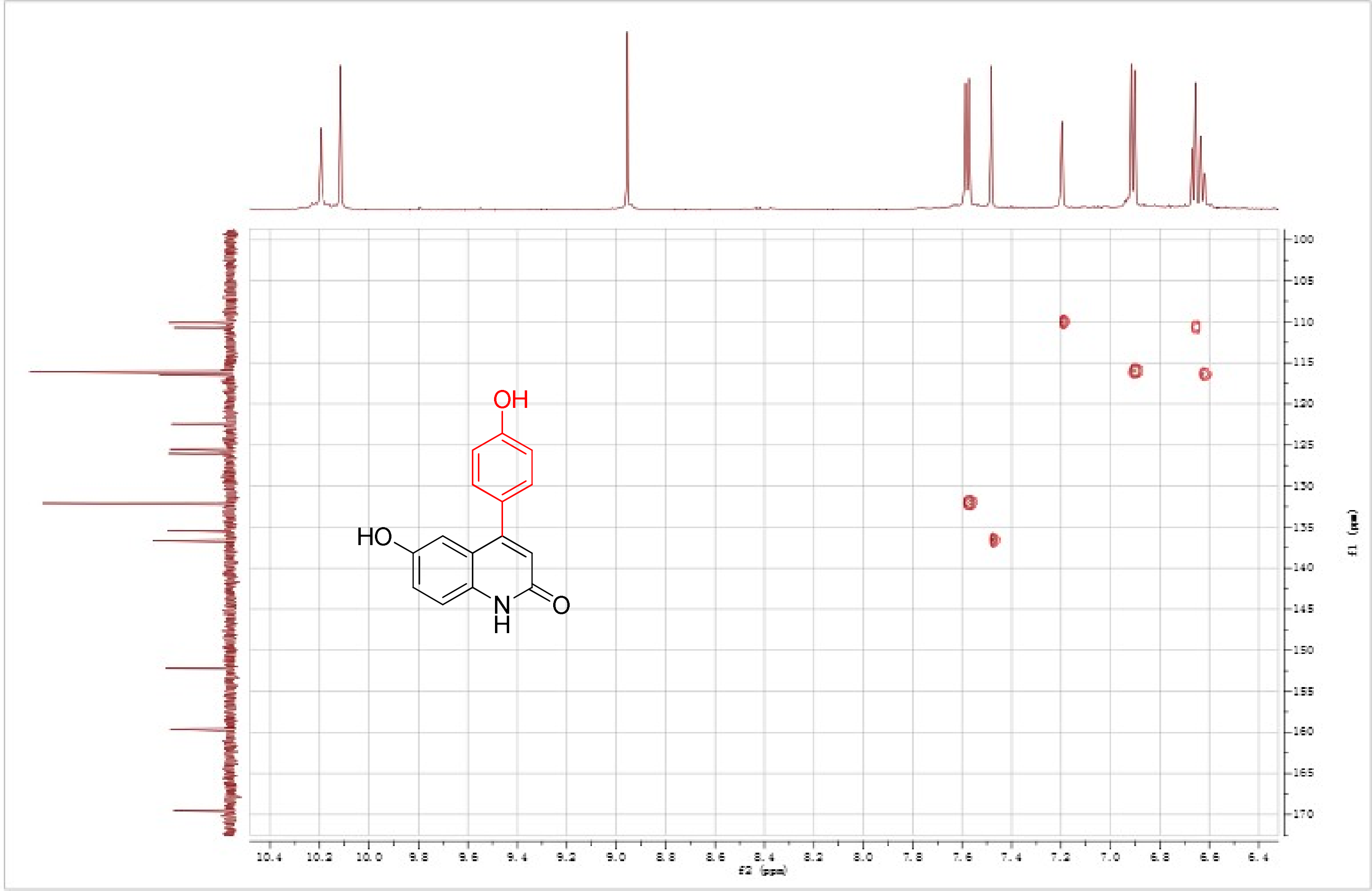

Figure S6. The HSQC spectrum of **1** (in DMSO-d_6)

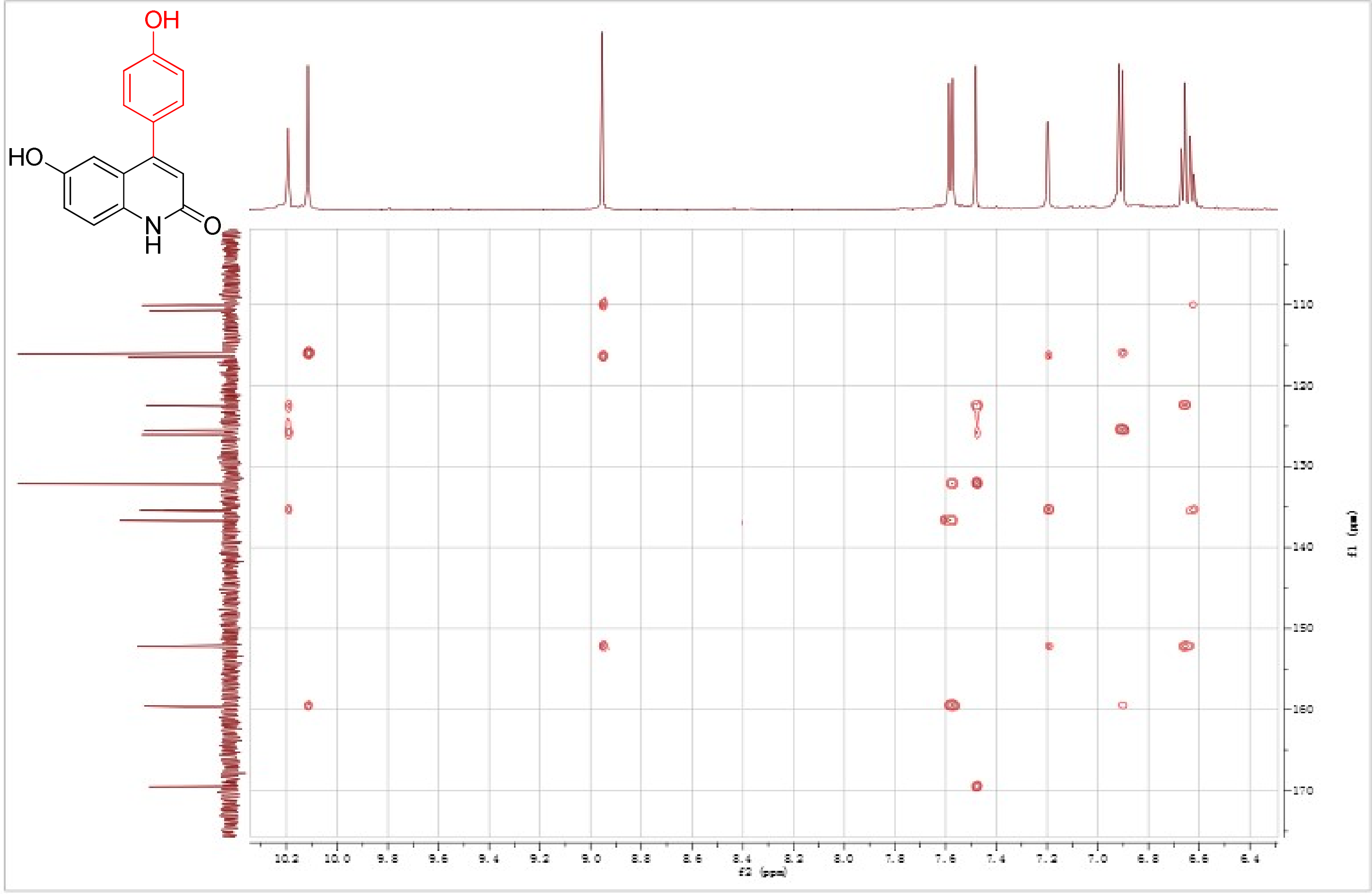

Figure S7. The HMBC spectrum of **1** (in DMSO-d_6)

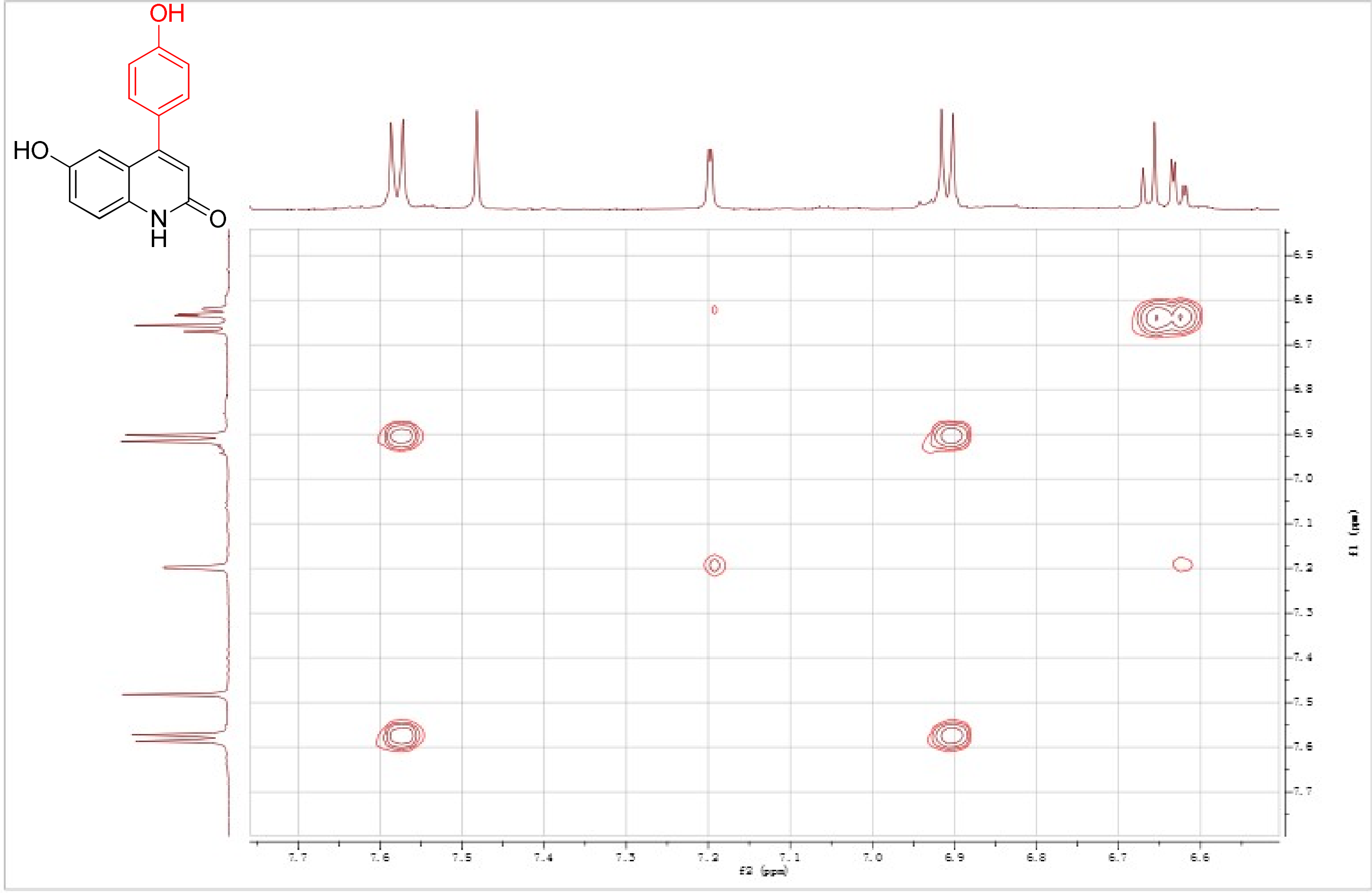

Figure S8. The ^{1}H-^{1}H COSY spectrum of **1** (in DMSO-d_6)

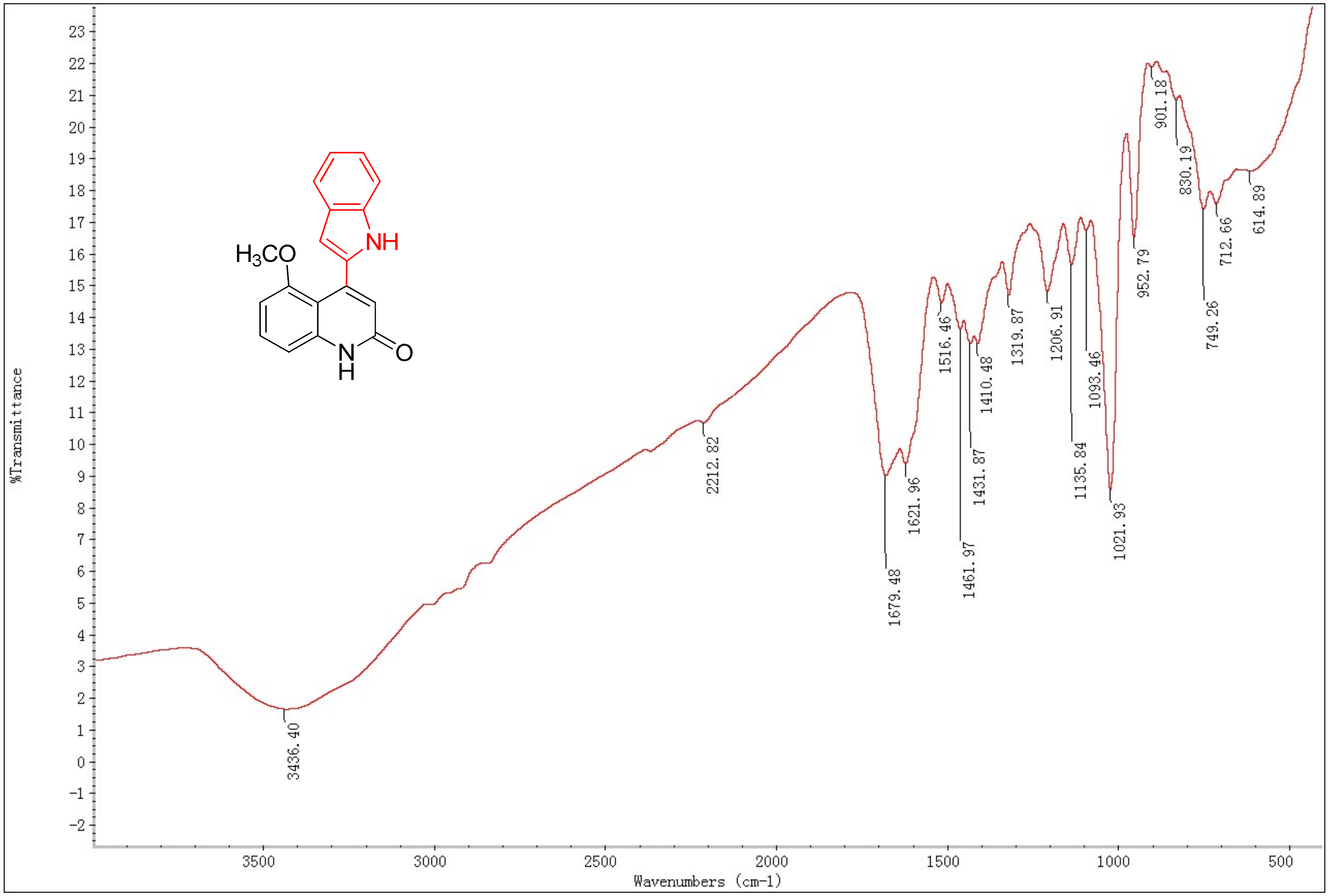

Figure S9. The IR spectrum of **2** (in KBr)

Qualitative Analysis Report

**Data Filename**	ESIH_20181116_ZWL_ZYT_19.d	**Sample Name**	ZYT-002-50
**Sample Type**	Sample	**Position**	P1-D2
**Instrument Name**	Agilent G6520 Q-TOF	**Acq Method**	20160322_MS_ESIH_POS_1min.m
**Acquired Time**	11/16/2018 11:20:49	**IRM Calibration Status**	Success
**DA Method**	small molecular data analysis method.m	**Comment**	ESIH by ZZY

User Spectra

Fragmentor Voltage	Collision Energy	Ionization Mode
110	0	ESI

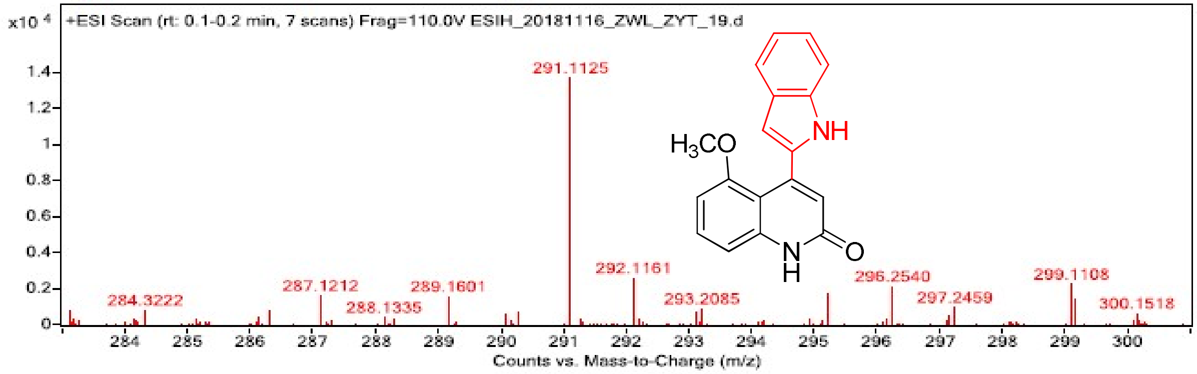

Formula Calculator Results

m/z	Calc m/z	Diff (mDa)	Diff (ppm)	Ion Formula	Ion
291.1125	291.1128	0.3	1.03	C18 H15 N2 O2	(M+H)+

--- End Of Report ---

Figure S10. The HR-ESI-MS spectrum of **2** (in MeOH)

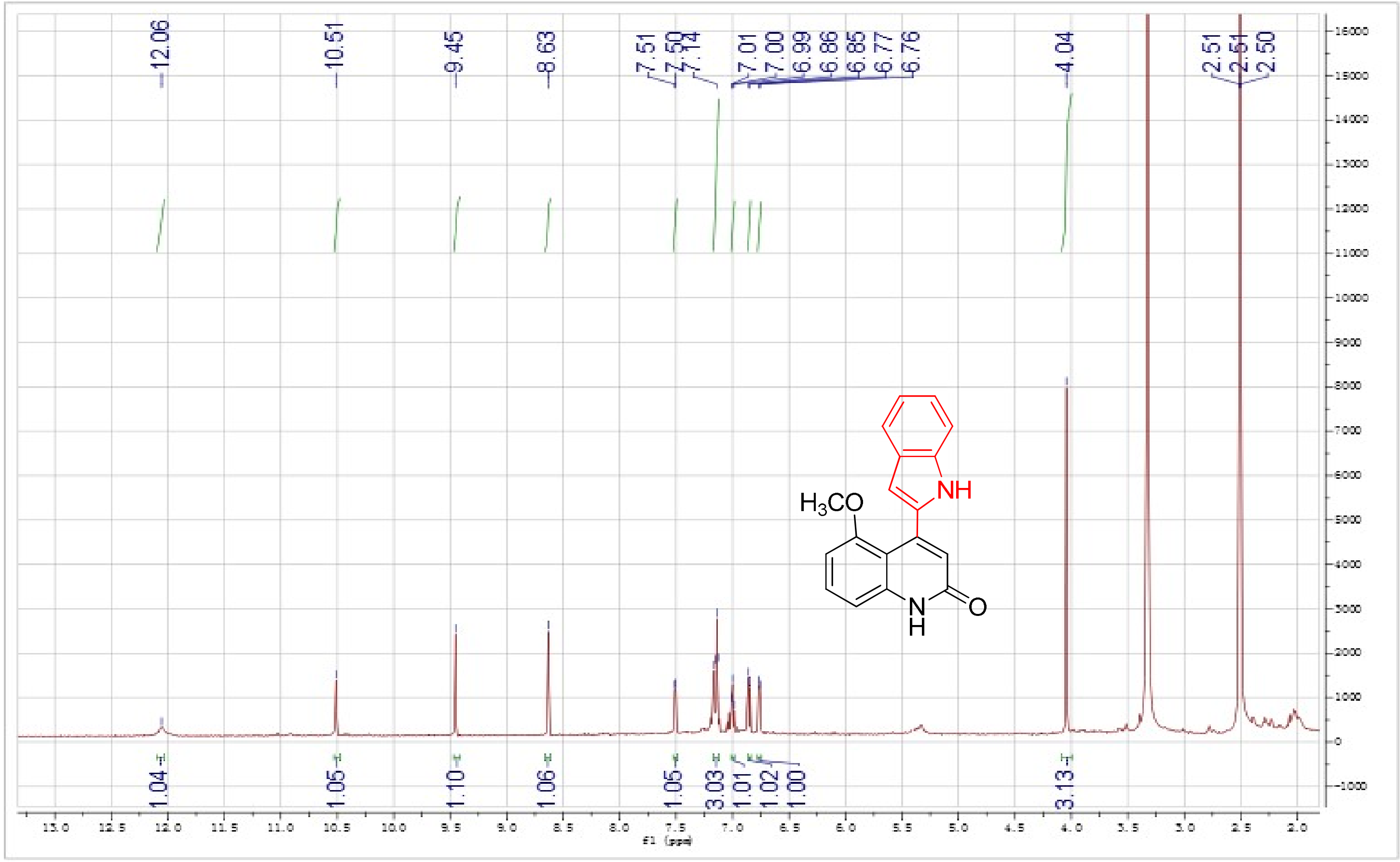

Figure S11. The ^{1}H NMR spectrum of **2** (in DMSO-d_6)

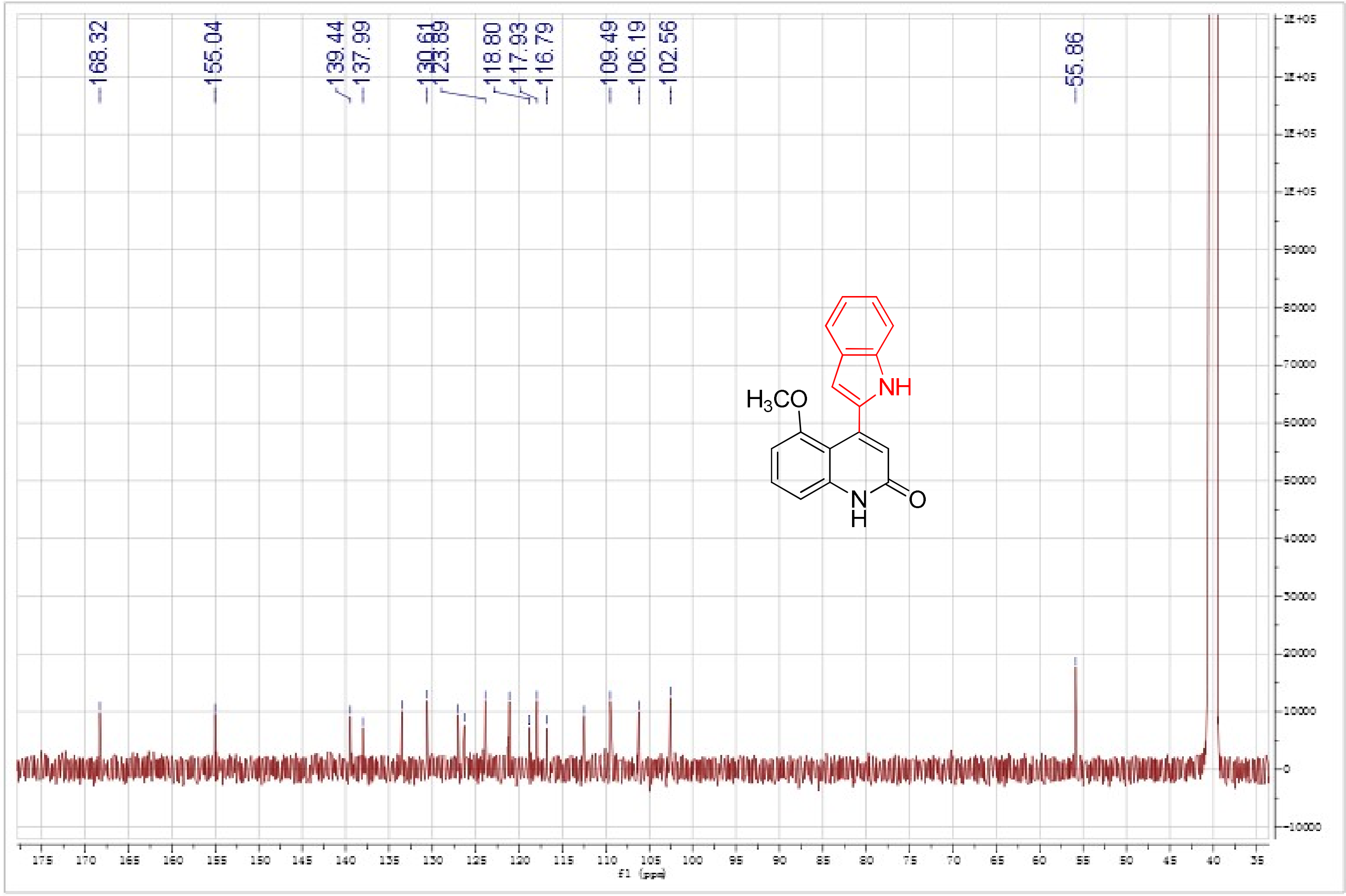

Figure S12. The ^{13}C NMR spectrum of **2** (in DMSO-d_6)

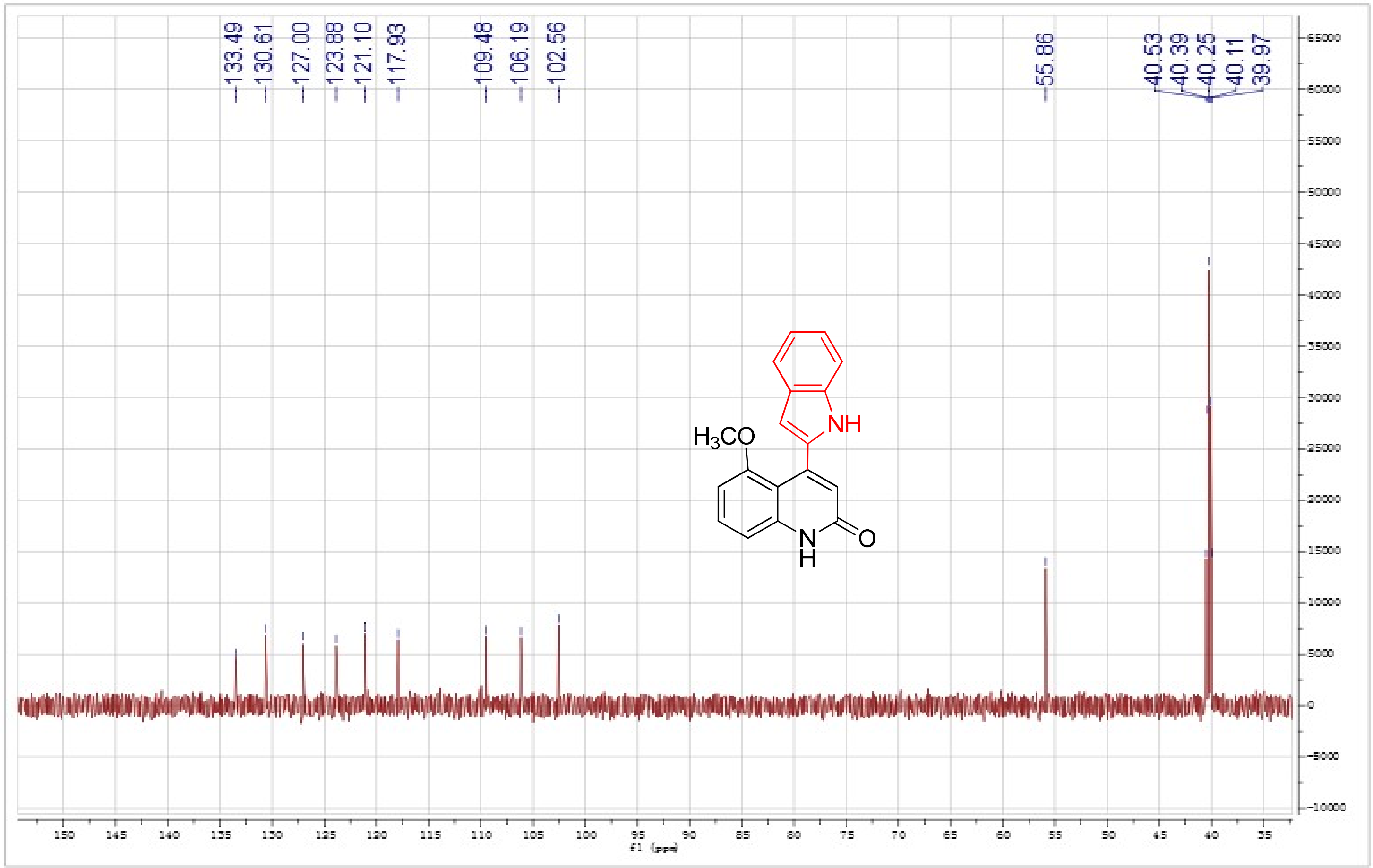

Figure S13. The DEPT 135 spectrum of **2** (in DMSO-d_6)

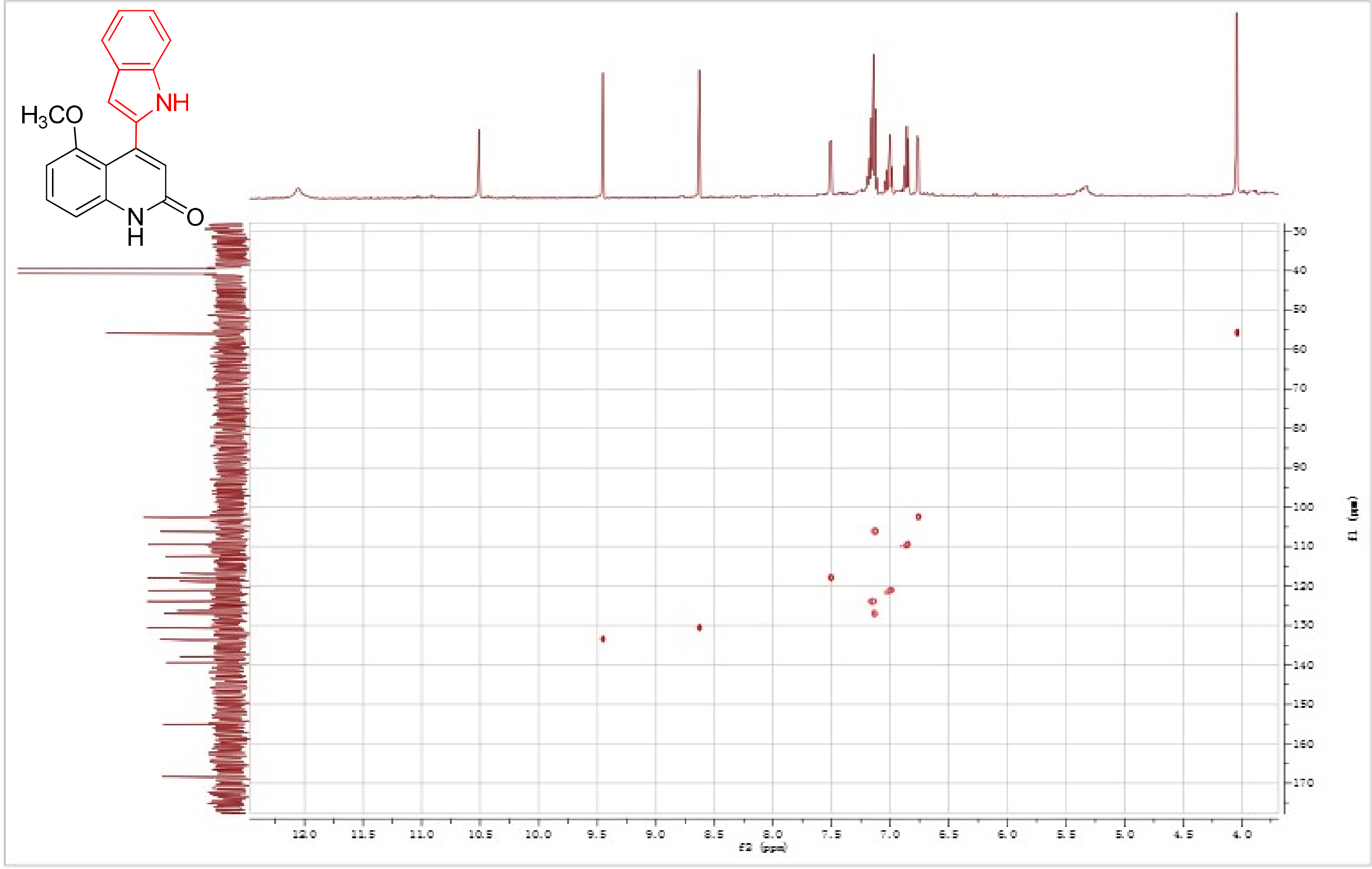

Figure S14. The HSQC spectrum of **2** (in DMSO-d_6)

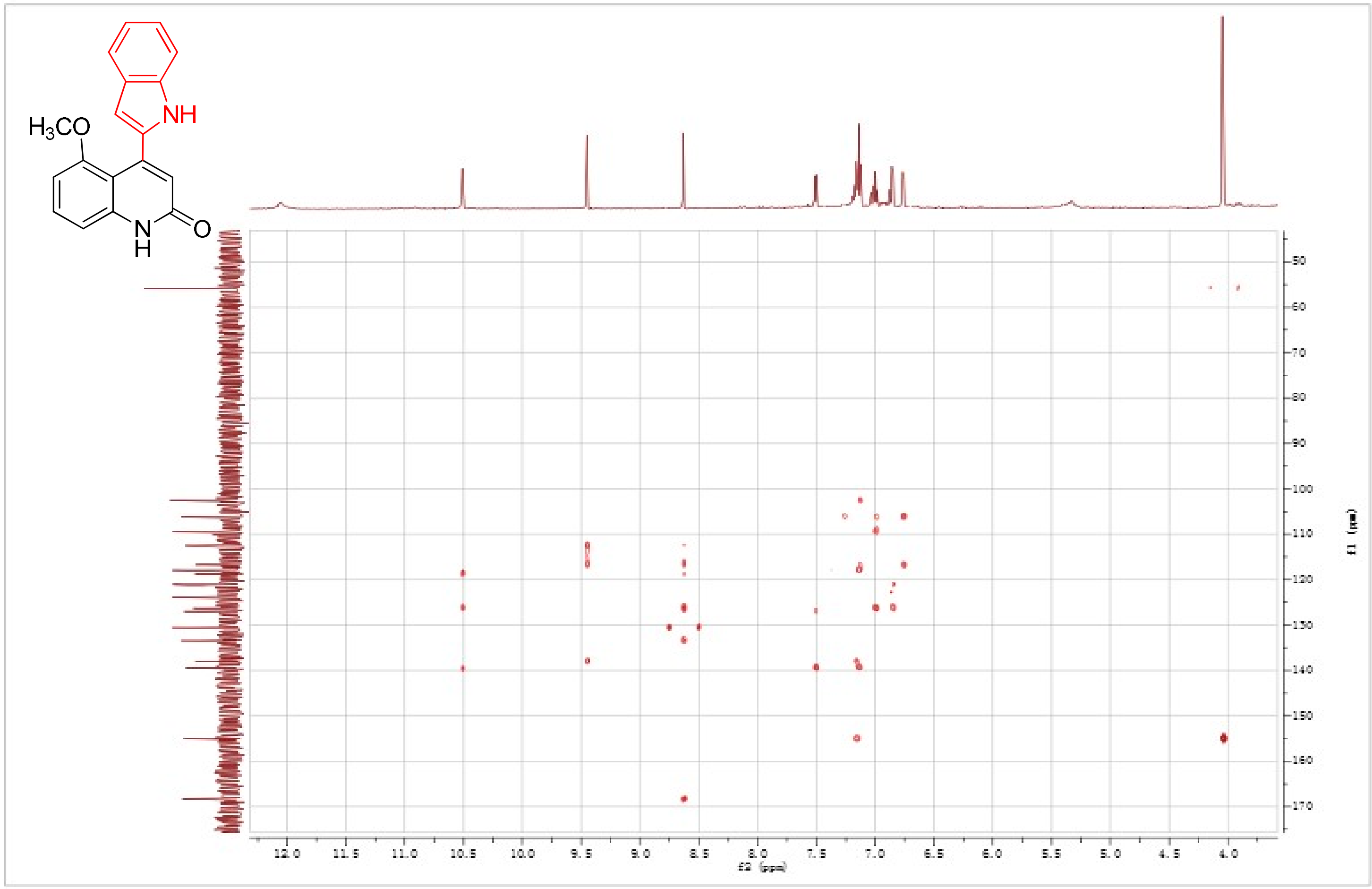

Figure S15. The HMBC spectrum of **2** (in DMSO-d_6)

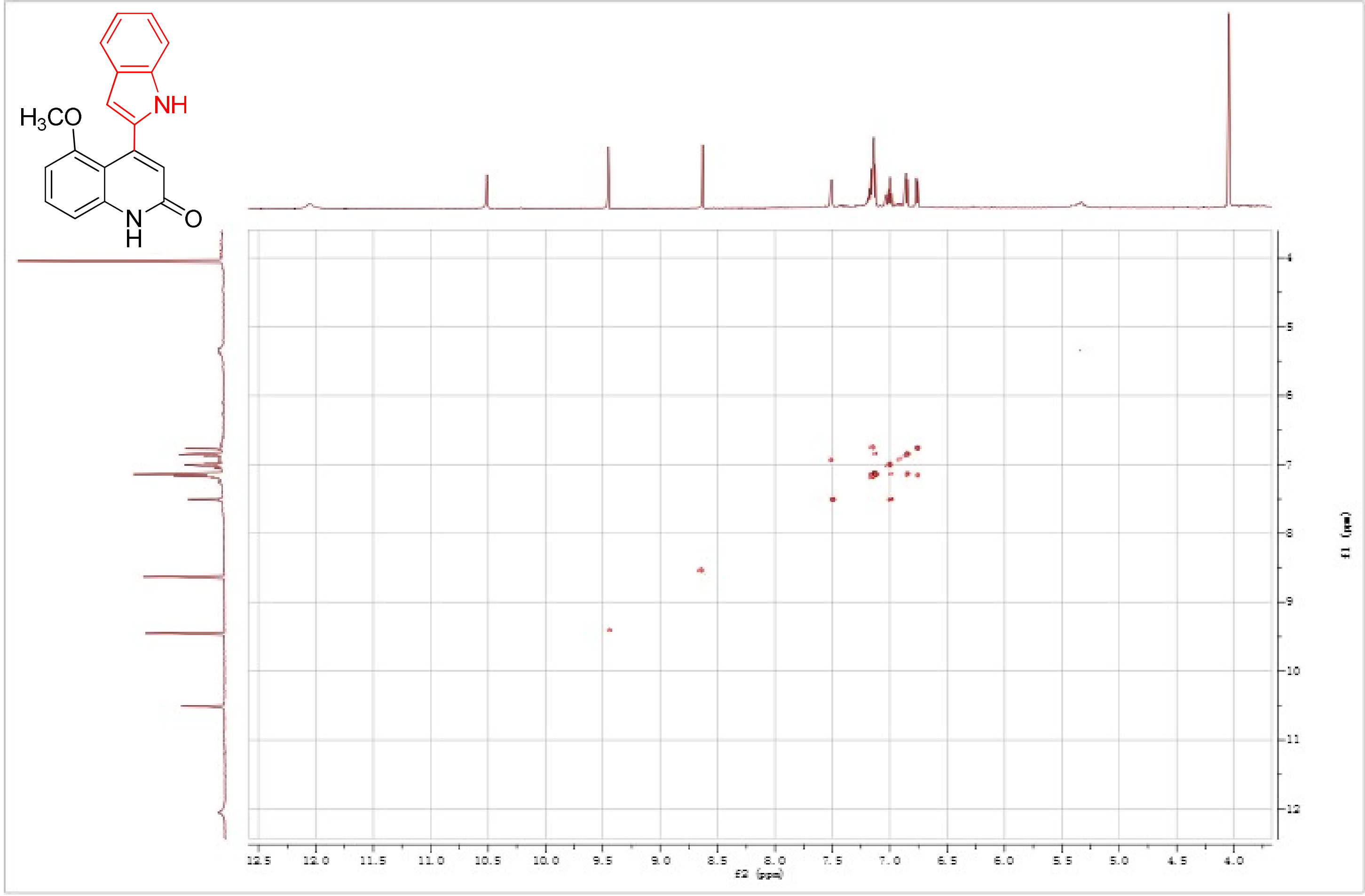

Figure S16. The ^{1}H-^{1}H COSY spectrum of **2** (in DMSO-d_6)

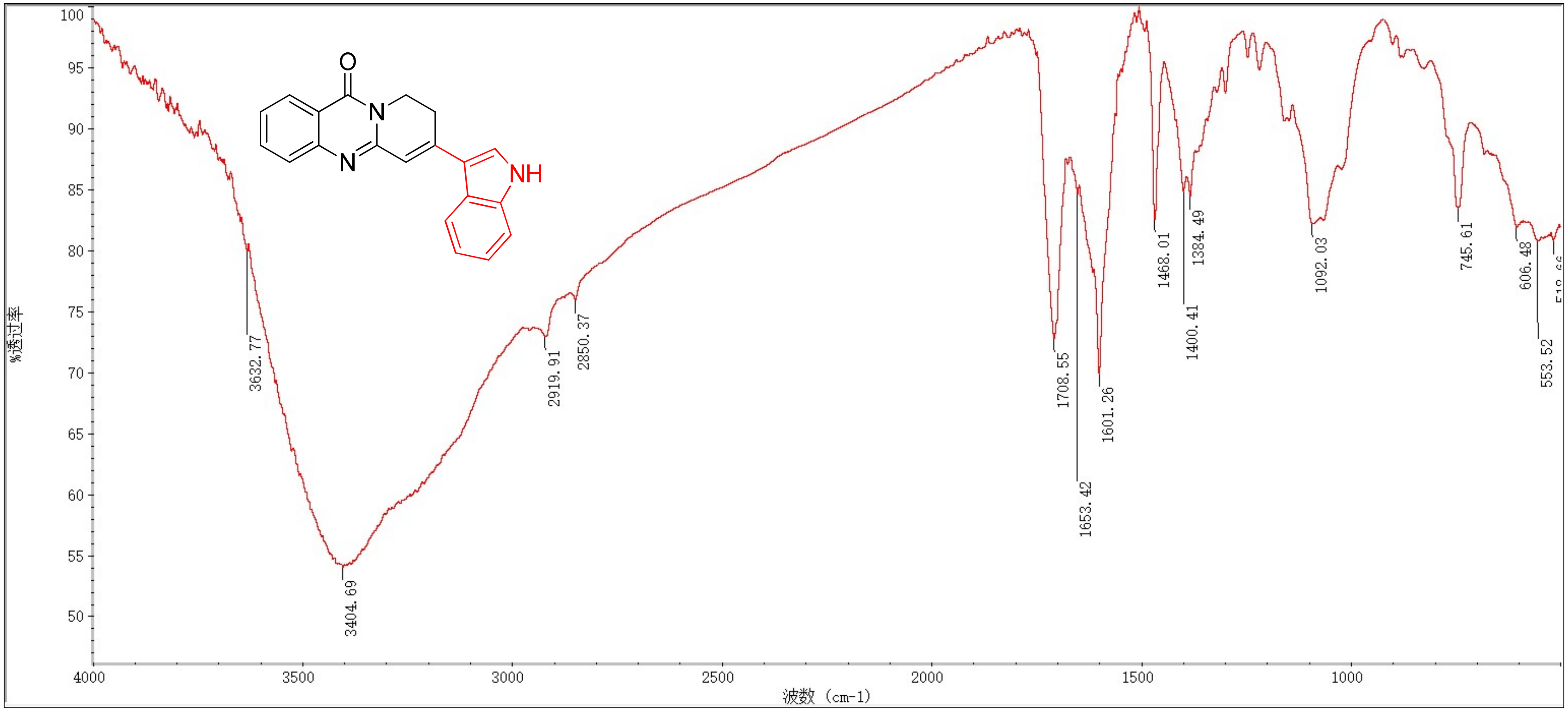

Figure S17. The IR spectrum of **3** (in KBr)

Qualitative Analysis Report

**Data Filename**	ESIH_20181116_ZWL_ZYT_17.d	**Sample Name**	ZYT-002-48
**Sample Type**	Sample	**Position**	P1-C9
**Instrument Name**	Agilent G6520 Q-TOF	**Acq Method**	20160322_MS_ESIH_POS_1min.m
**Acquired Time**	11/16/2018 11:17:06	**IRM Calibration Status**	Success
**DA Method**	small molecular data analysis method.m	**Comment**	ESIH by ZZY

User Spectra

Fragmentor Voltage	Collision Energy	Ionization Mode
110	0	ESI

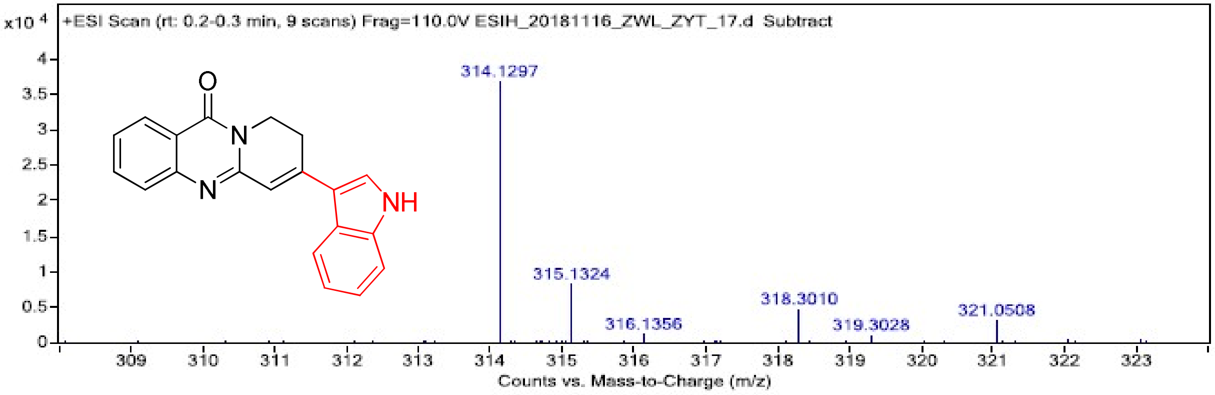

Formula Calculator Results

m/z	Calc m/z	Diff (mDa)	Diff (ppm)	Ion Formula	Ion
314.1297	314.1288	-0.92	-2.93	C20 H16 N3 O	(M+H)+

--- End Of Report ---

Figure S18. The HR-ESI-MS spectrum of **3** (in MeOH)

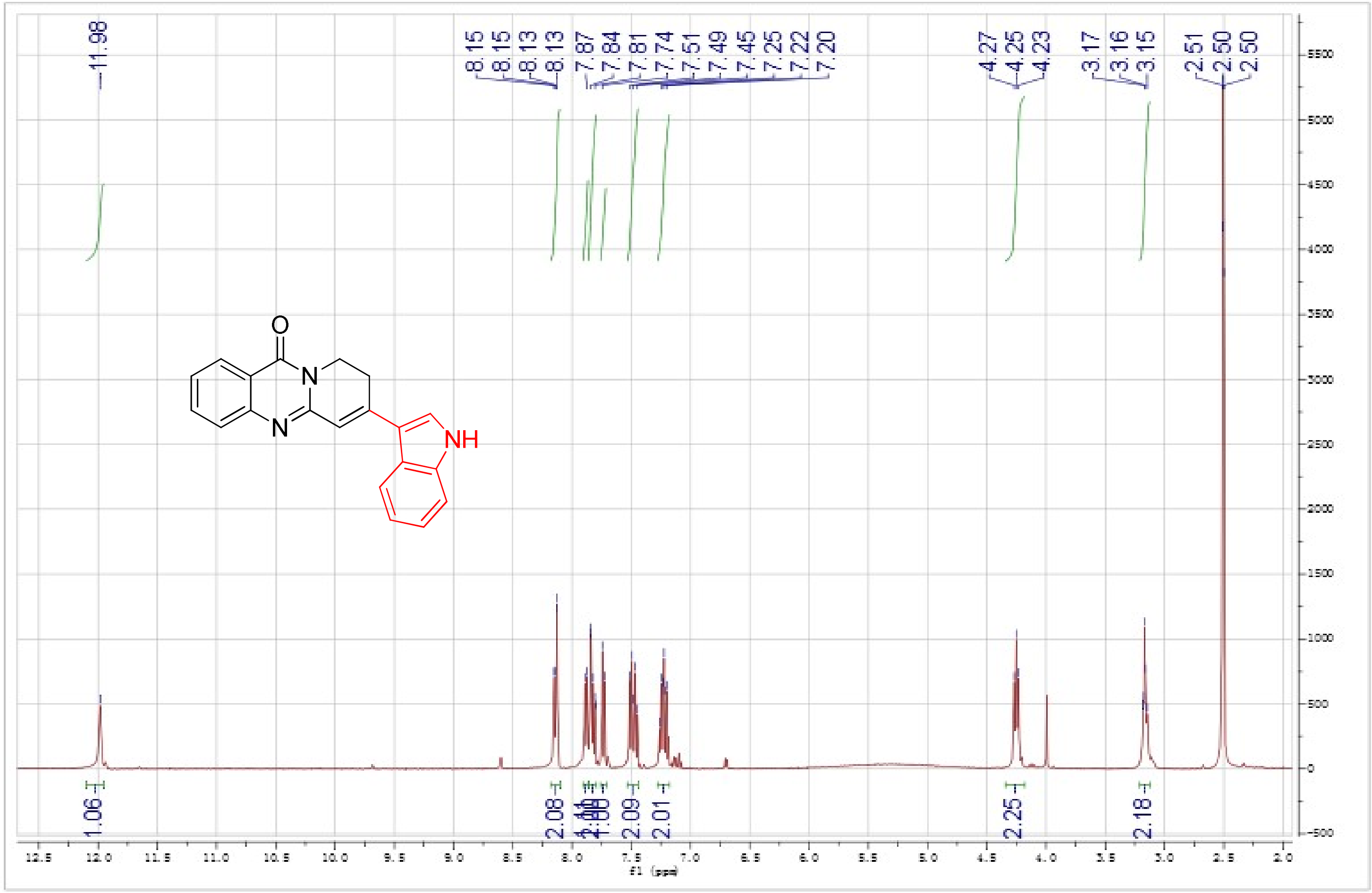

Figure S19. The ^{1}H NMR spectrum of **3** (in DMSO-d_6)

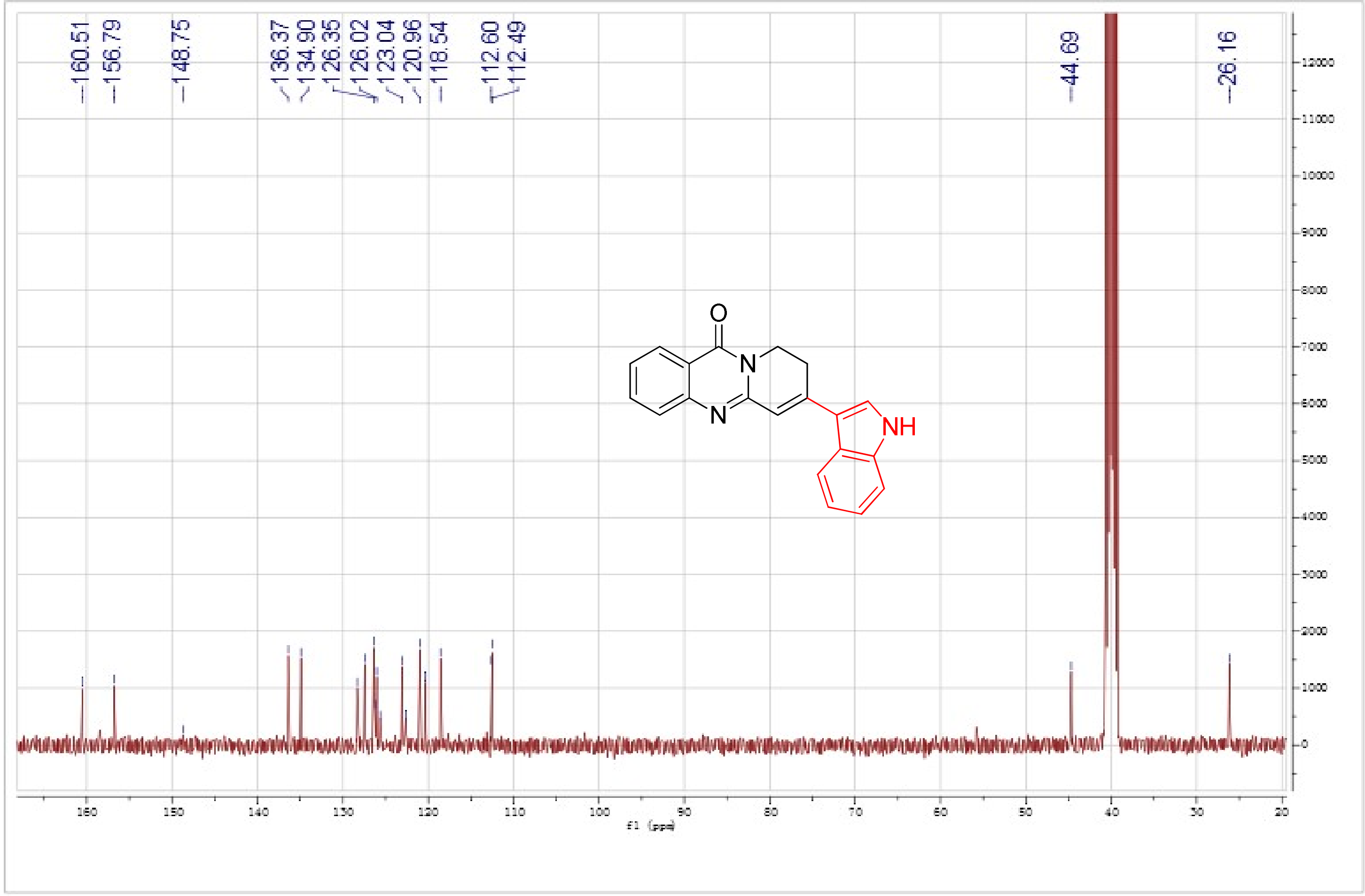

Figure S20. The ^{13}C NMR spectrum of **3** (in DMSO-d_6)

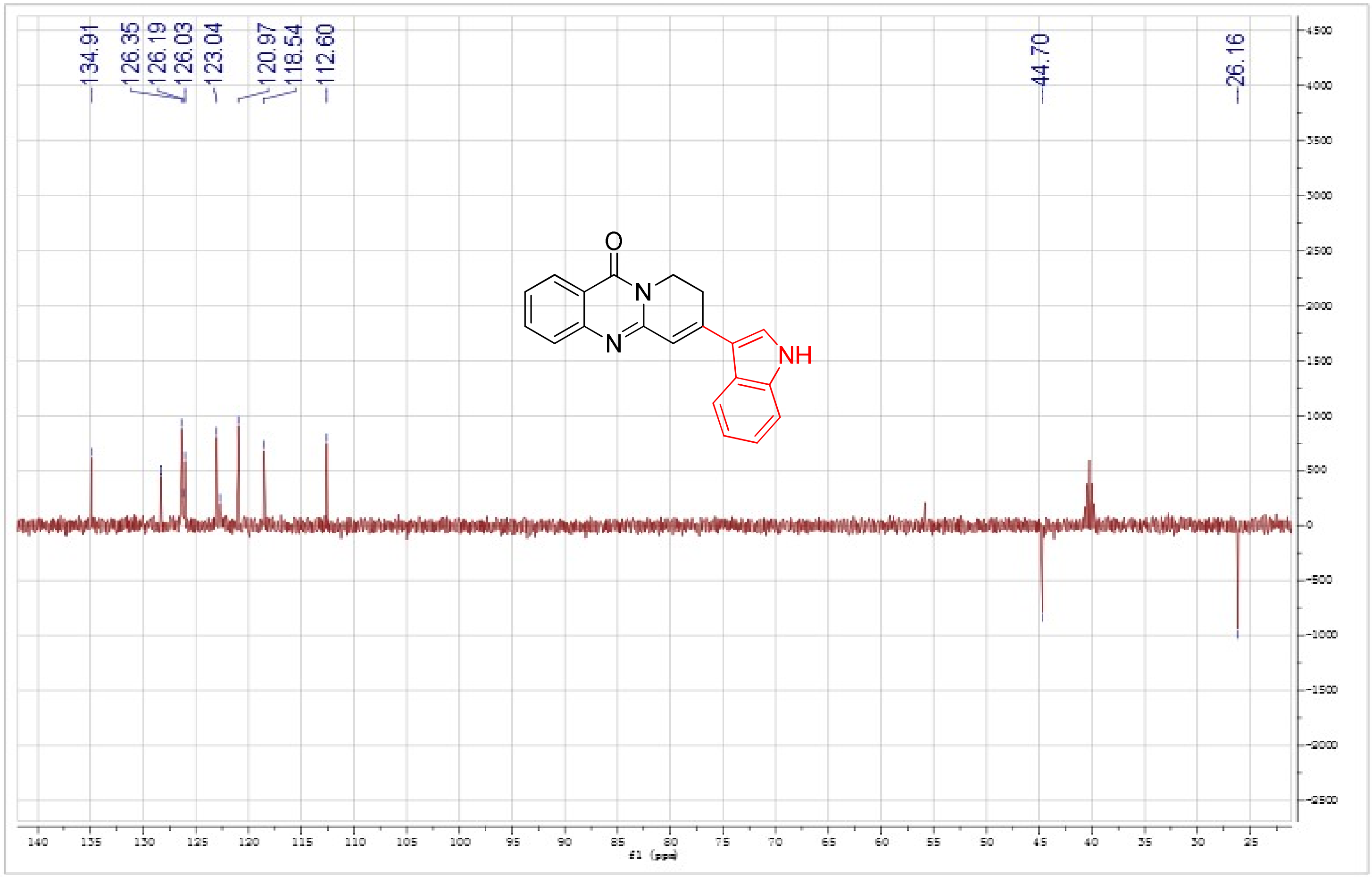

Figure S21. The DEPT 135 spectrum of **3** (in DMSO-d_6)

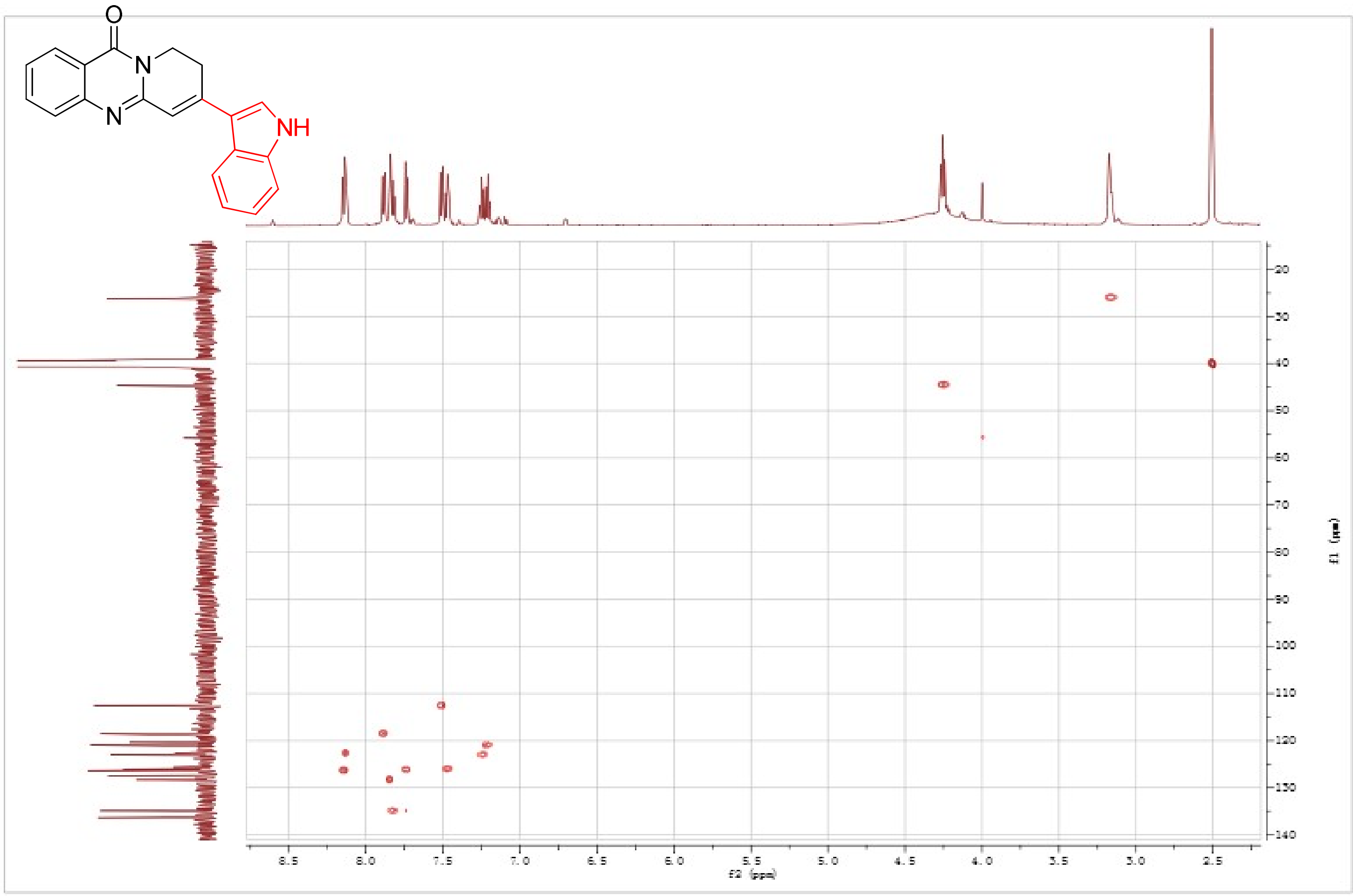

Figure S22. The HSQC spectrum of **3** (in DMSO-d_6)

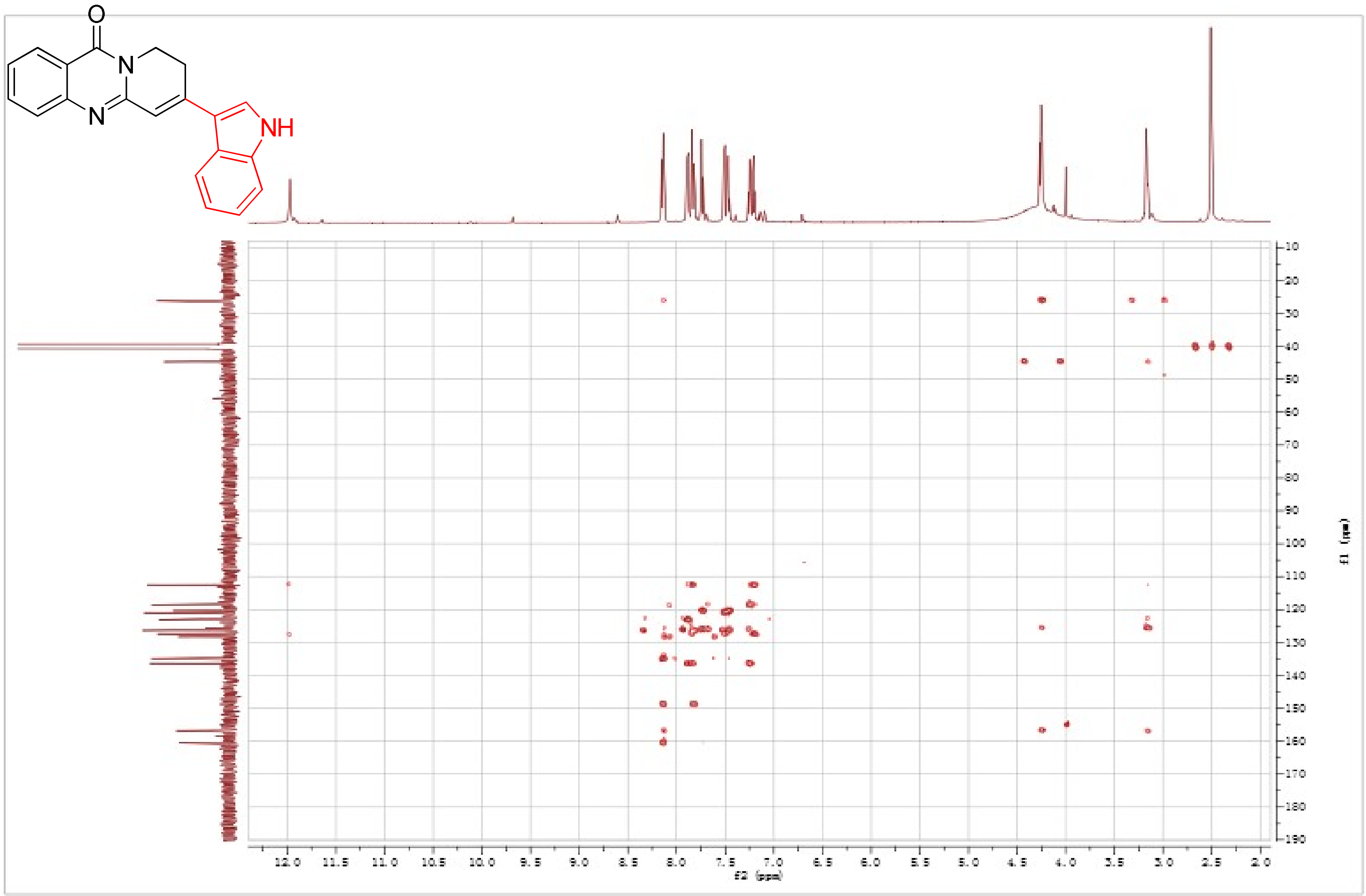

Figure S23. The HMBC spectrum of **3** (in DMSO-d_6)

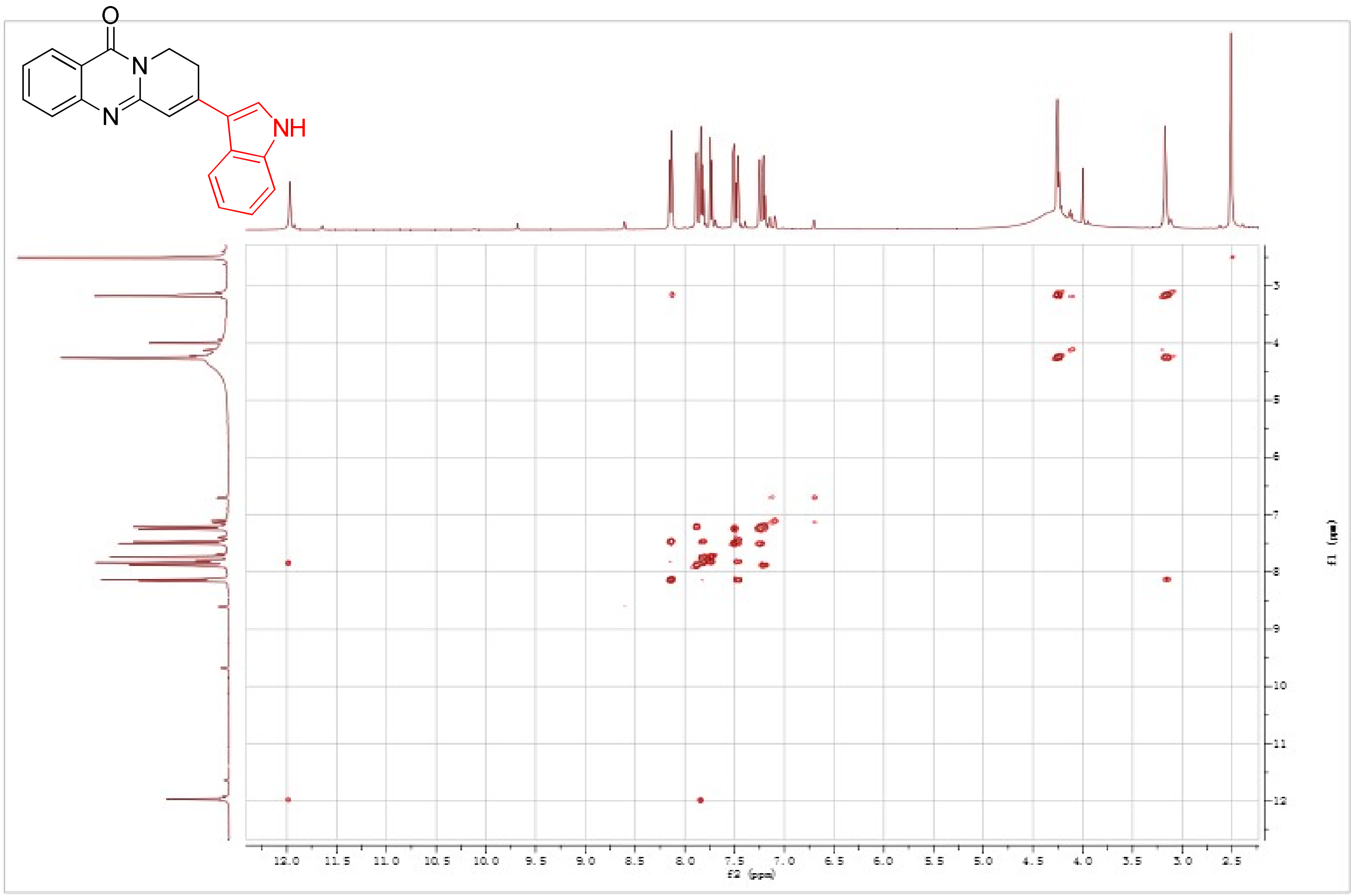

Figure S24. The ^{1}H-^{1}H COSY spectrum of **3** (in DMSO-d_6)

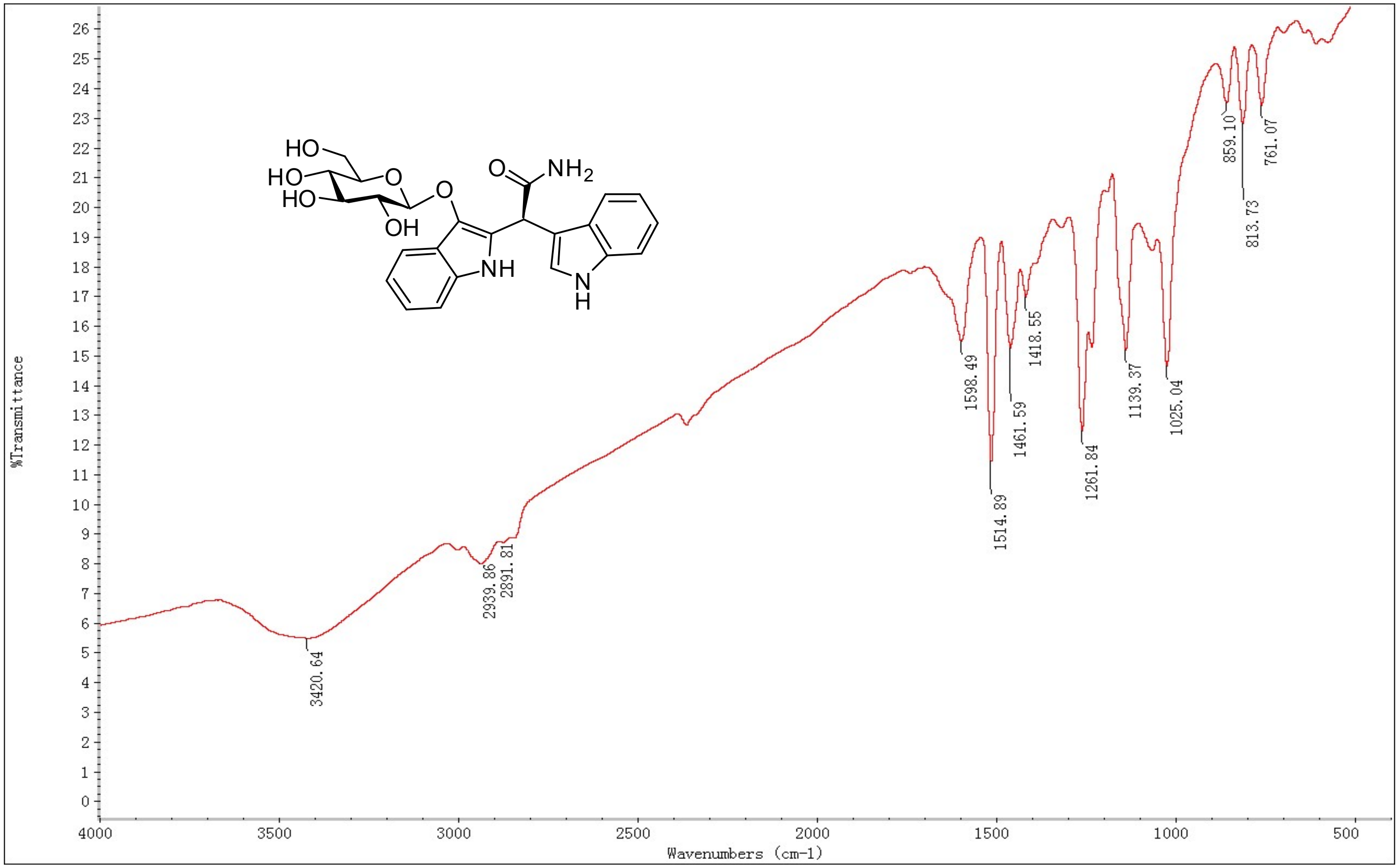

Figure S25. The IR spectrum of **23** (in KBr)

Qualitative Analysis Report

**Data Filename**	ESIH_20181126_ZWL_ZYT_31.d	**Sample Name**	ZYT-002-52
**Sample Type**	Sample	**Position**	P1-D2
**Instrument Name**	Agilent G6520 Q-TOF	**Acq Method**	20160322_MS_ESIH_POS_1min.m
**Acquired Time**	11/26/2018 11:43:01	**IRM Calibration Status**	Success
**DA Method**	small molecular data analysis method.m	**Comment**	ESIH by ZZY

User Spectra

Fragmentor Voltage	Collision Energy	Ionization Mode
175	0	ESI

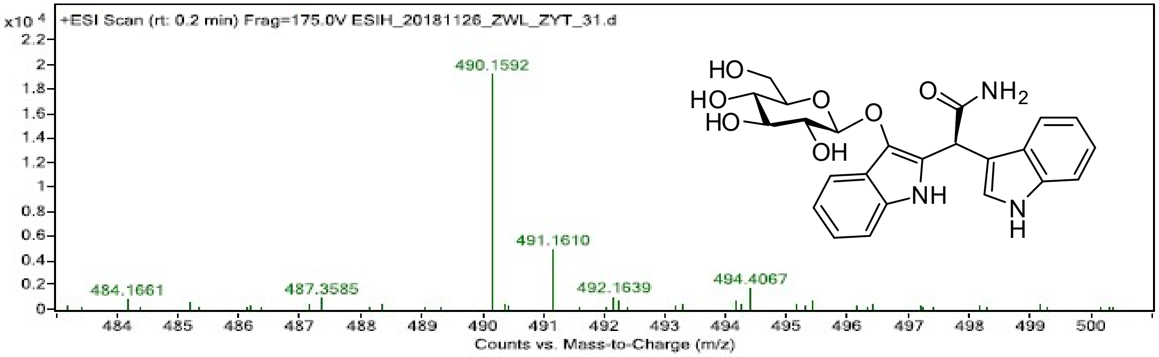

Formula Calculator Results

m/z	Calc m/z	Diff (mDa)	Diff (ppm)	Ion Formula	Ion
490.1592	490.1585	-0.7	-1.43	C24 H25 N3 Na O7	(M+Na)+

--- End Of Report ---

Agilent Technologies Page 1 of 1 Printed at: 15:42 on: 11/26/2018

Figure S26. The HR-ESI-MS spectrum of **23** (in MeOH)

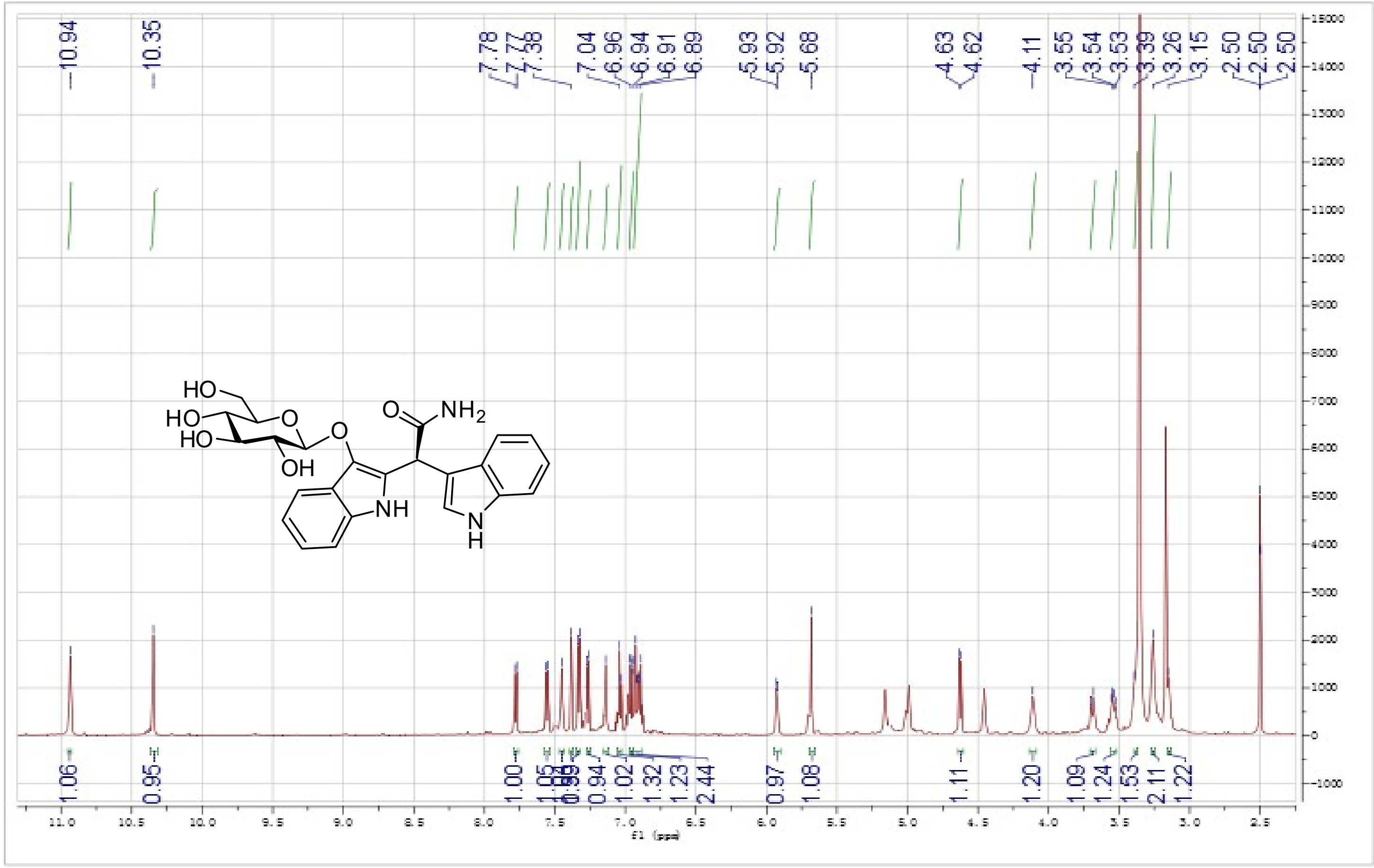

Figure S27. The ^1H NMR spectrum of **23** (in DMSO-d_6)

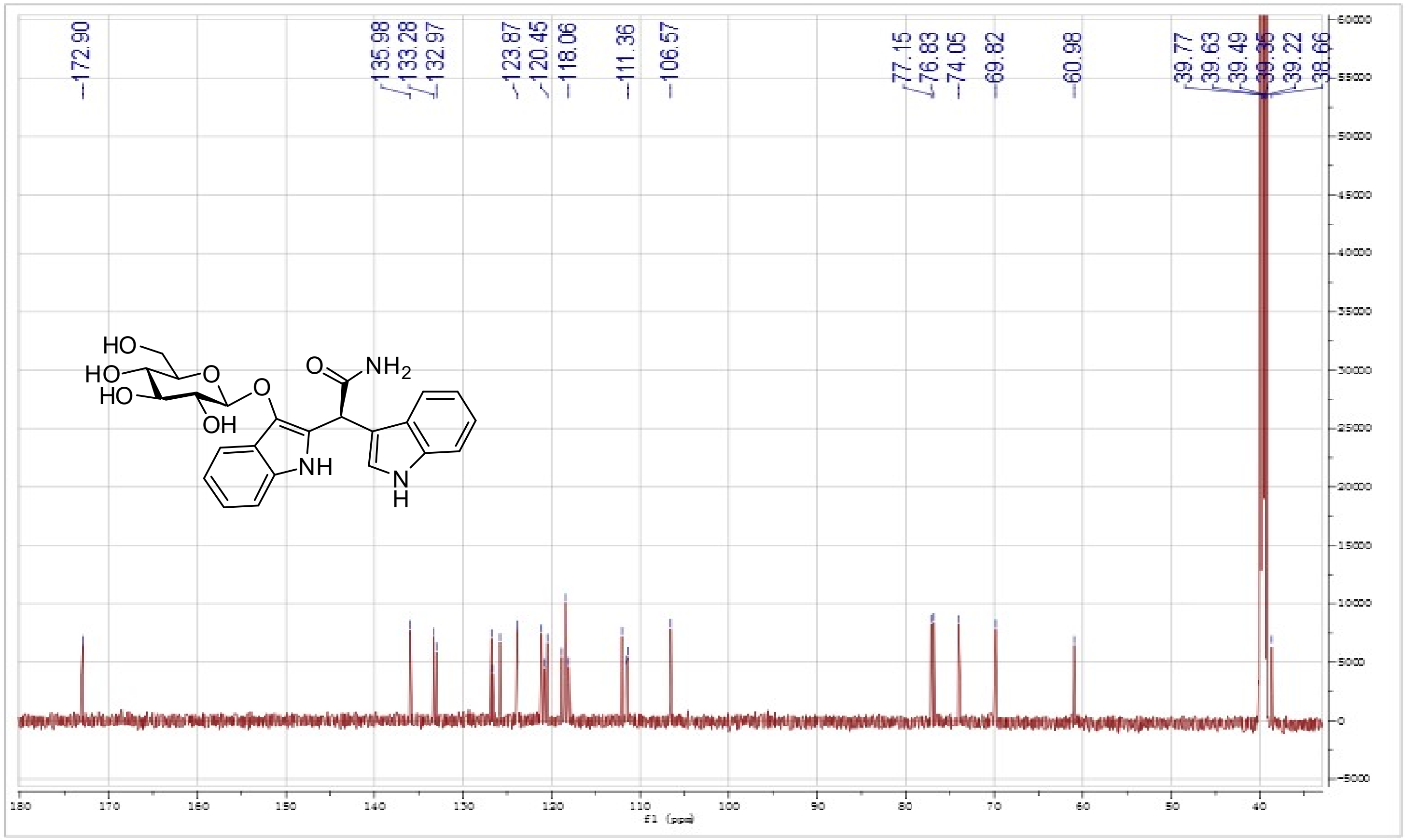

Figure S28. The ^{13}C NMR spectrum of **23** (in DMSO-d_6)

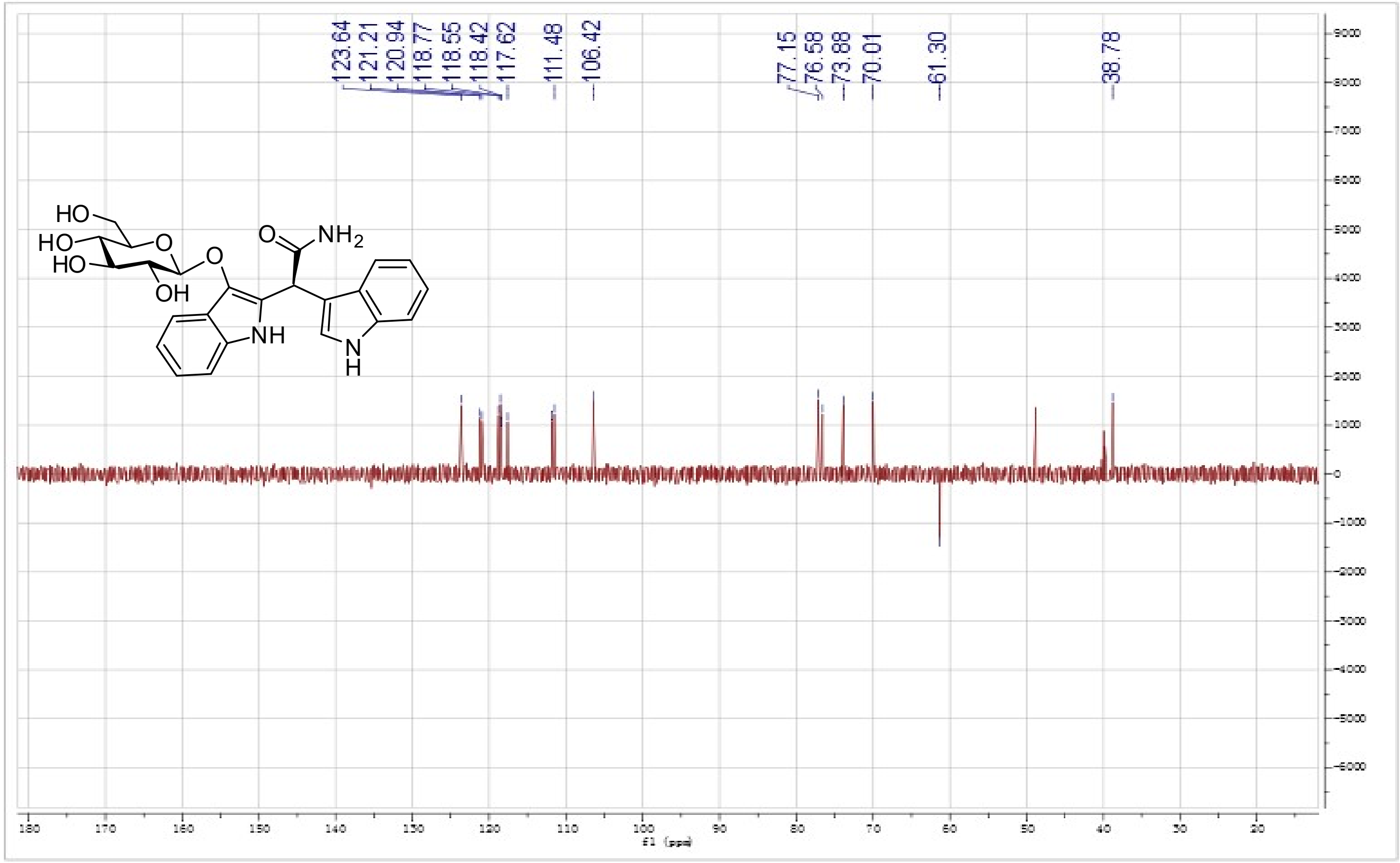

Figure S29. The DEPT 135° spectrum of **23** (in DMSO-d_6)

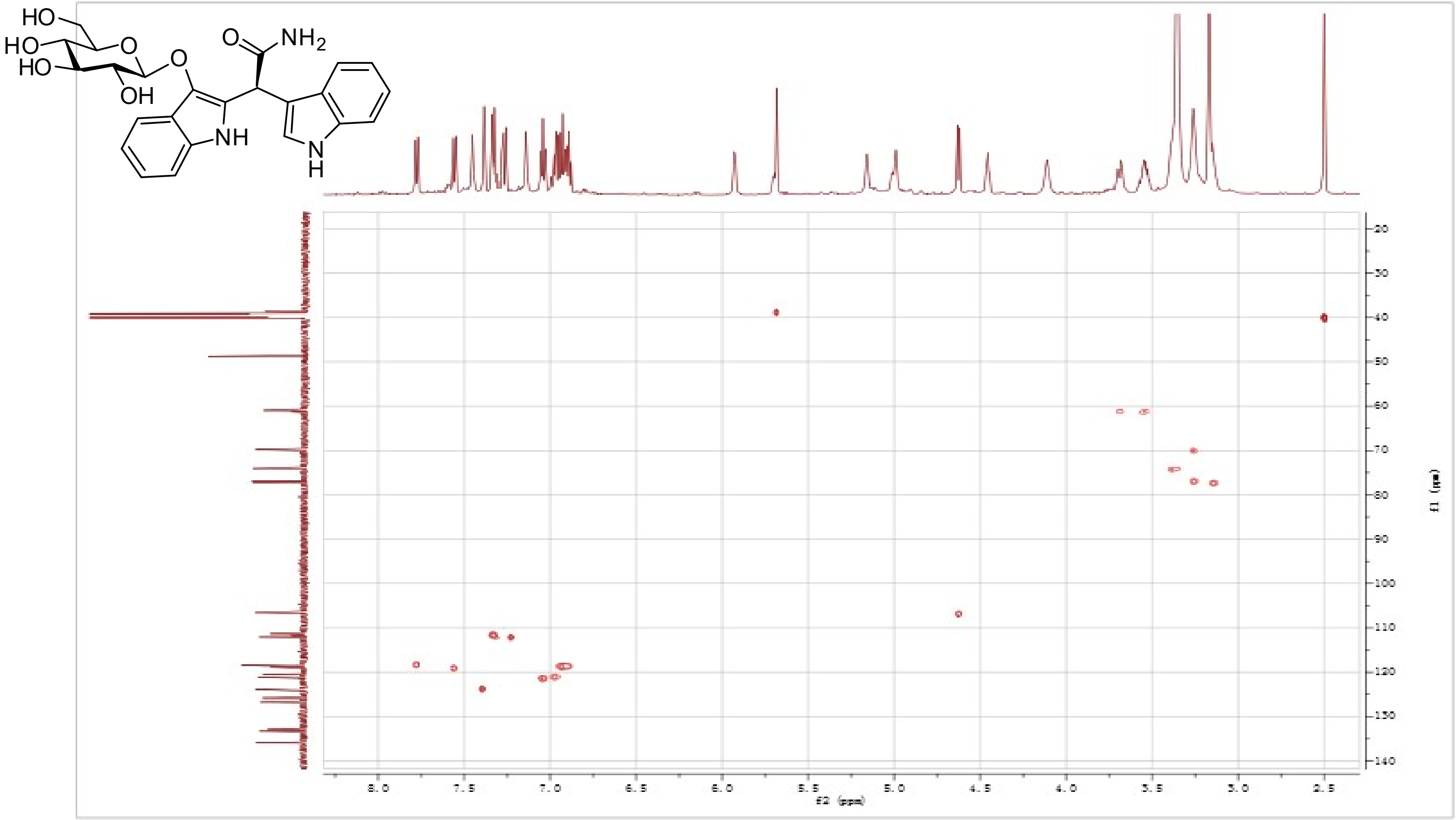

Figure S30. The HSQC spectrum of **23** (in DMSO-d_6)

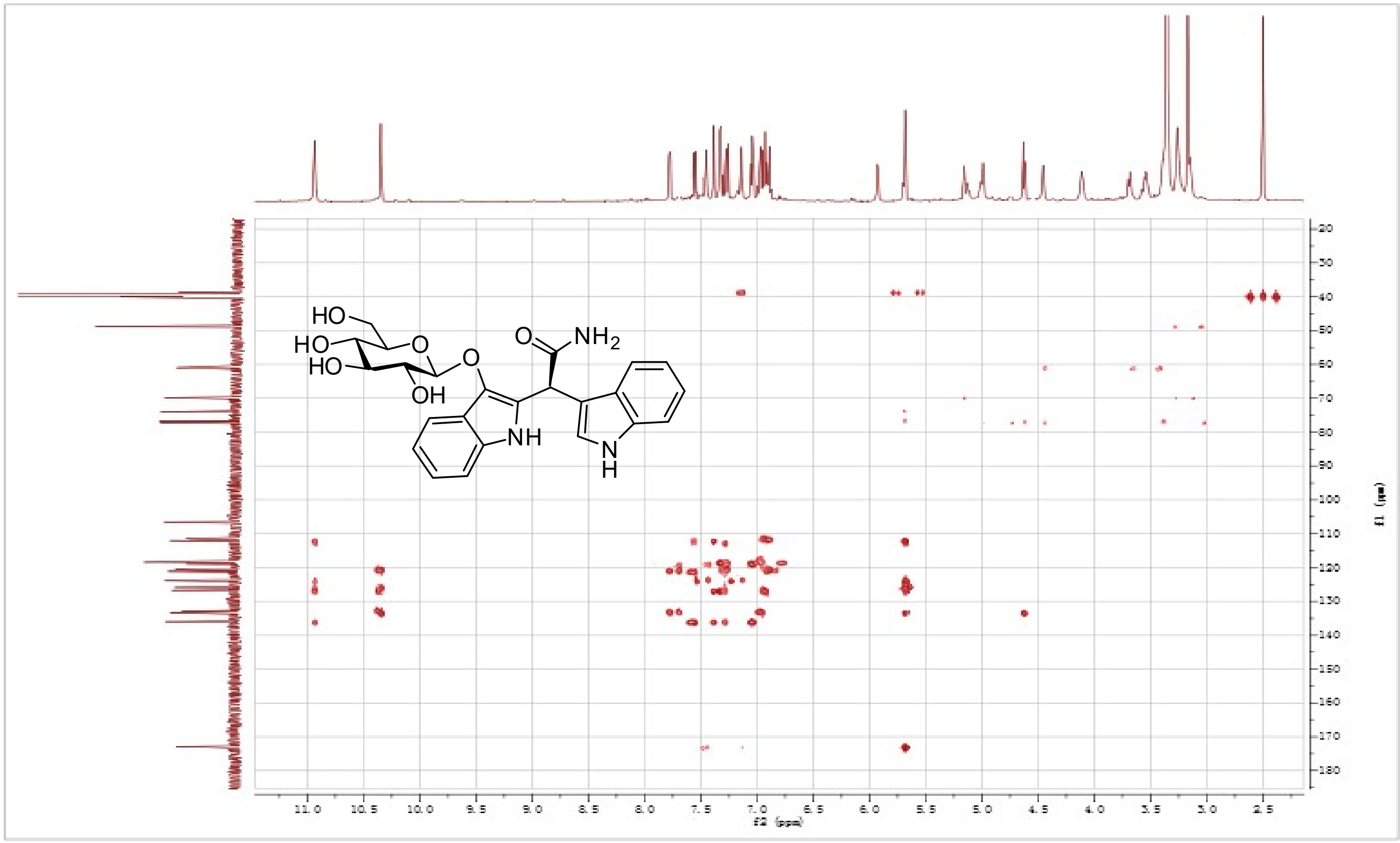

Figure S31. The HMBC spectrum of **23** (in DMSO-d_6)

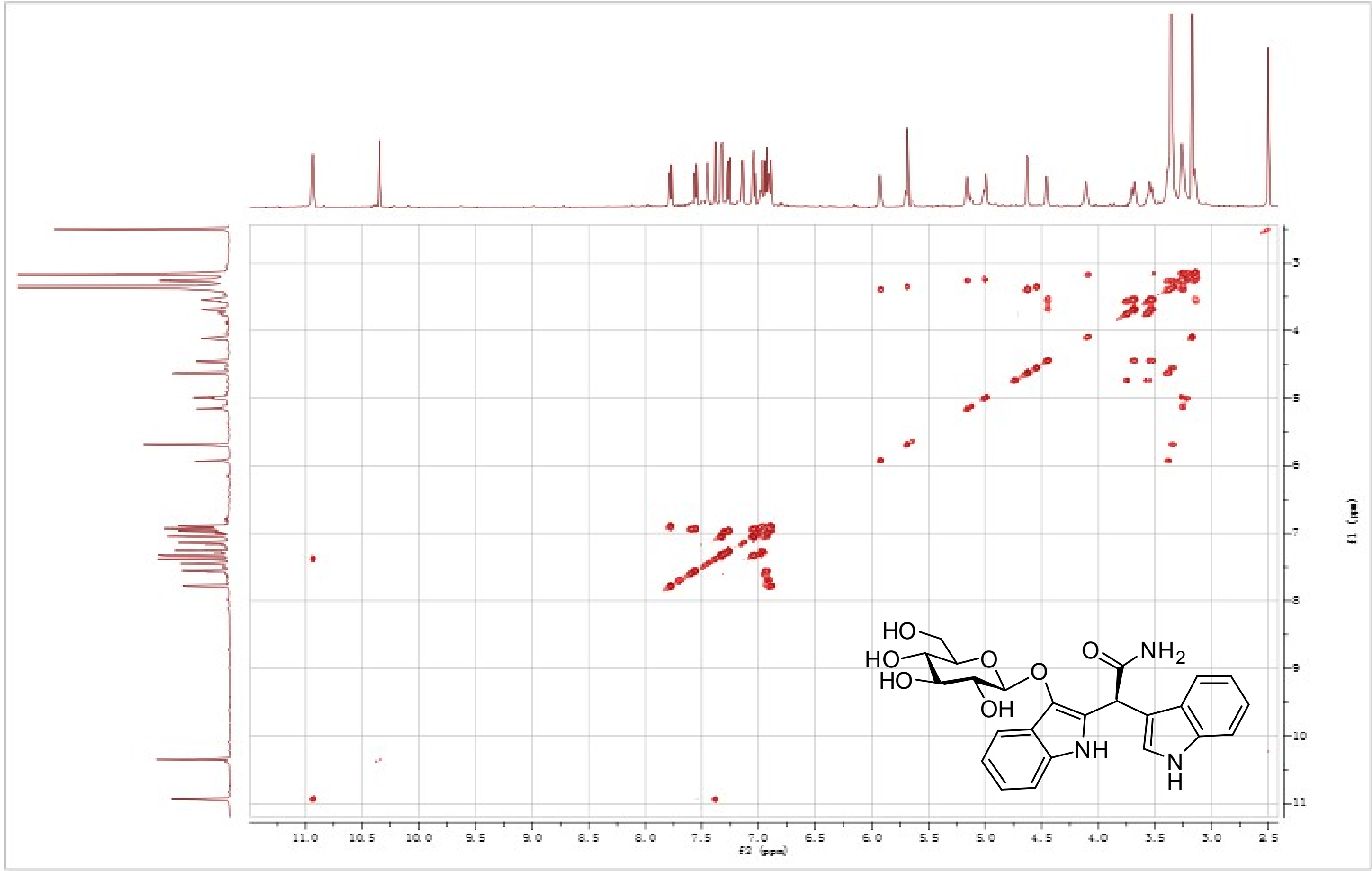

Figure S32. The ^{1}H-^{1}H COSY spectrum of **23** (in DMSO-d_6)

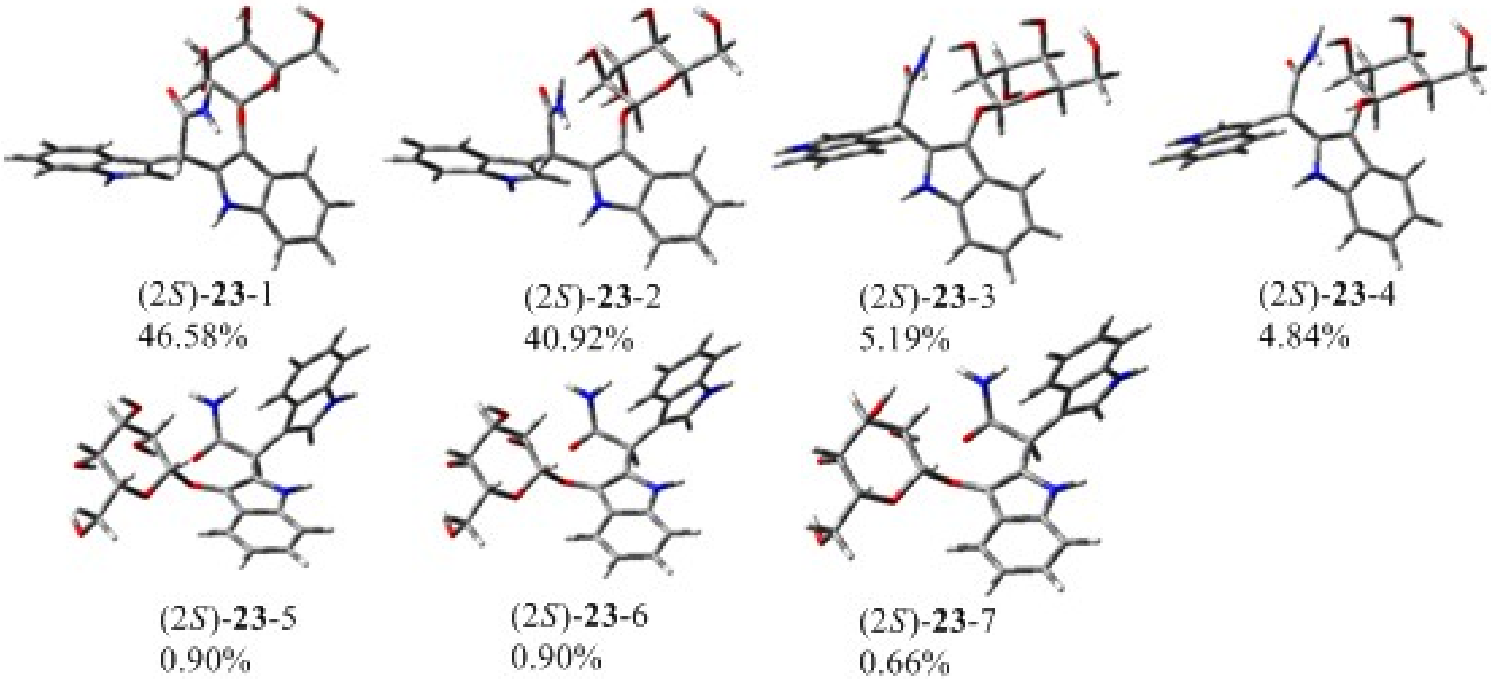

Figure S33. b3lyp/6-31g(d) optimized lowest energy conformers for (2*S*)-**23** and their equilibrium populations

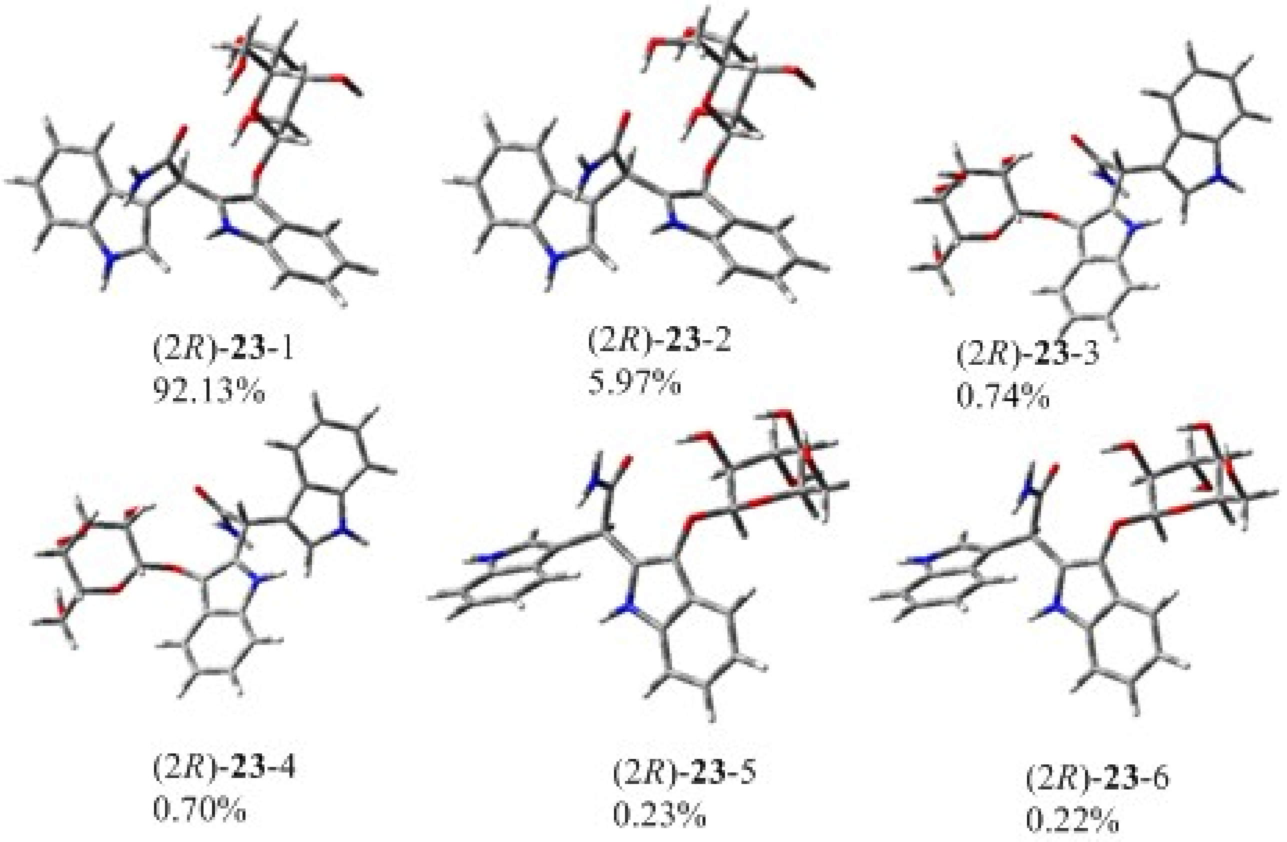

Figure S34. b3lyp/6-31g(d) optimized lowest energy conformers for (2*R*)-**23** and their equilibrium populations

The experimental ECD spectrum of **23** (red line) and the calculated ECD spectrum of (2)-**23** (red short dash) and (2*R*)-**23** (blue short dash). The calculated ECD (excited states 30) spectrum were plotted as sums of Gaussians 09 with a 0.20 eV exponential half-width using the program Specdis 1.62, and the UV shifted was 11 nm.

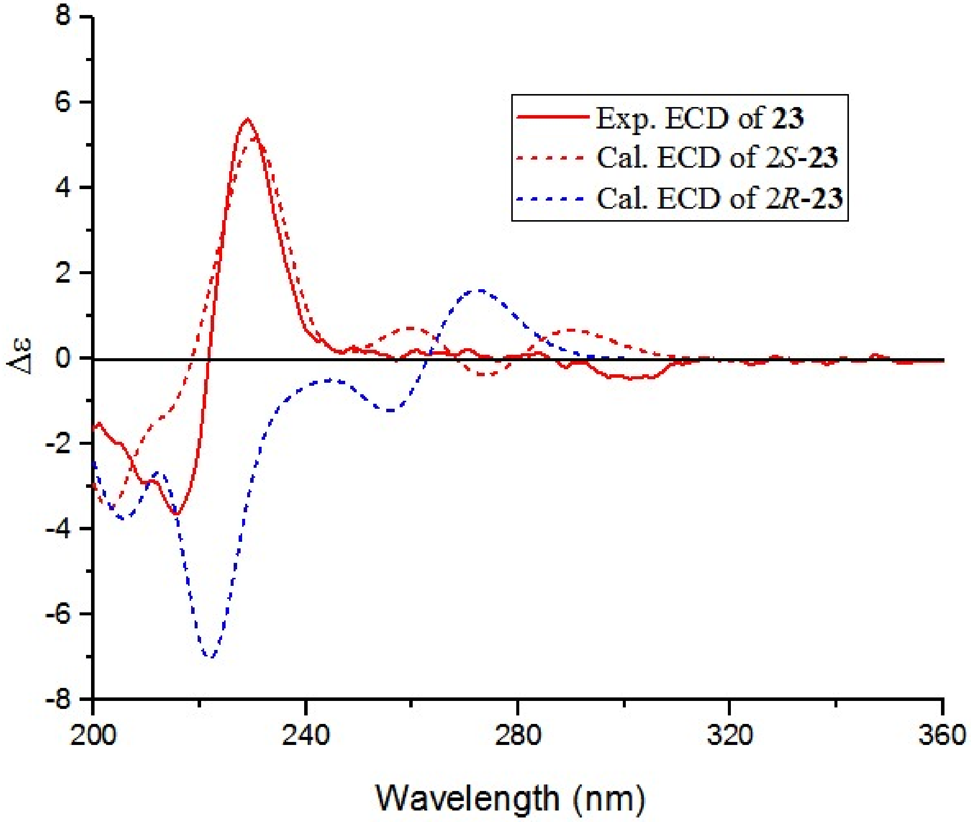

Figure S35. Experimental and calculated ECD spectrum of **23**
